# Modeling the Conformational Changes Underlying Channel Opening in CFTR

**DOI:** 10.1371/journal.pone.0074574

**Published:** 2013-09-27

**Authors:** Kazi S. Rahman, Guiying Cui, Stephen C. Harvey, Nael A. McCarty

**Affiliations:** 1 Petit Institute of Bioengineering and Bioscience and School of Biology, Georgia Institute of Technology, Atlanta, Georgia, United States of America; 2 Department of Pediatrics and Center for Cystic Fibrosis Research, Emory University and Children's Healthcare of Atlanta, Inc., Atlanta, Georgia, United States of America; Universidad Autonoma de San Luis Potosi, Mexico

## Abstract

Mutations in the gene encoding the cystic fibrosis transmembrane conductance regulator protein (CFTR) cause cystic fibrosis (CF), the most common life-shortening genetic disease among Caucasians. Although general features of the structure of CFTR have been predicted from homology models, the conformational changes that result in channel opening and closing have yet to be resolved. We created new closed- and open-state homology models of CFTR, and performed targeted molecular dynamics simulations of the conformational transitions in a channel opening event. The simulations predict a conformational wave that starts at the nucleotide binding domains and ends with the formation of an open conduction pathway. Changes in side-chain interactions are observed in all major domains of the protein, and experimental confirmation was obtained for a novel intra-protein salt bridge that breaks near the end of the transition. The models and simulation add to our understanding of the mechanism of ATP-dependent gating in this disease-relevant ion channel.

## Introduction

CFTR, the protein defective in Cystic Fibrosis (CF), is a large transmembrane protein belonging to the ATP-binding cassette (ABC) transporter superfamily [1], [2]. This ATP-gated chloride ion channel plays a central role in ion and water movement across epithelia [3], [4]. Loss of CFTR function leads to the impaired mucociliary clearance and consequent chronic airway infection seen in CF patients [5], whereas excessive enterotoxin-mediated activation of CFTR can result in secretory diarrhea [6].

Characteristic of its ABC transporter heritage [2], [7], CFTR has a domain architecture comprising two membrane-spanning domains (MSD1 and MSD2), each containing six transmembrane α-helices (enumerated TM1-TM12), and two cytosolic nucleotide binding domains (NBD1 and NBD2). Unique among ABC transporters, CFTR also has a cytosolic regulatory (R) domain that is the primary site of protein kinase A-mediated phosphorylation, required for channel activation.

In most ABC transporters, ATP binding at the two NBDs results in the formation of a head-to-tail NBD dimer that drives a conformational change in the whole protein which enables membrane transport [8]. Unlike most other ABC proteins, however, CFTR shows significant disparity in both the sequence and function of the two NBDs. Whereas the second composite ATP-binding site (formed at the interface between the Walker motifs in NBD2 and the signature sequence in NBD1) is capable of both ATP binding and hydrolysis, the first composite ATP-binding site (formed at the interface between the Walker motifs in NBD1 and the signature sequence in NBD2) is thought to have poor hydrolytic activity, engaging in stable interactions that retain the nucleotide at the binding site [9]. Conformational transitions in CFTR are therefore thought to involve at least four distinct states: 1) C0: an apo closed-channel state where both nucleotide-binding sites are empty and the NBDs are completely dissociated; 2) C1: a closed-channel state where the first ATP-binding site is occupied and the NBDs are partially dimerized; 3) C2: a strained transition state where the NBDs are fully dimerized but the channel is still closed to conduction; and 4) O: an open-channel state with tightly dimerized NBDs in which both of the binding sites contain ATP.

CFTR is unique in being the only ABC protein known to serve as an ion channel, rather than exclusively as a transporter that might function by means of an “alternating access” cycle where the substrate binding pocket is alternately exposed to the cytoplasmic and extracellular spaces [10]. The structural basis for this remarkable departure in function in CFTR has not yet been fully elucidated. While high-resolution X-ray crystal structures of the nucleotide binding domains exist [11]–[13], structural information on the MSDs and the unique-to-CFTR R domain has been scarce, with only low-resolution NMR and cryo-EM electron density maps currently available [14], [15]. However, since ATP binding and tight NBD dimerization are necessary for channel opening [16], it has been hypothesized that CFTR evolved from a degraded ABC exporter whose ATP-bound outward-facing state (equivalent to the state in which the substrate binding pocket faces the extracellular space) corresponds to the open CFTR channel [17]. Due to the low ATP turnover rate at NBD1, the dominant closed channel conformation is thought to be the partial-dimer C1 state, although the fully dissociated C0 state is also likely to be closed to ion conduction.

The elucidation of the complete structure of the bacterial multidrug transporter Sav1866 in an outward-facing pose [18] led to its use as a template for several homology models of the CFTR open channel state [19]–[24]. Due to low sequence homology between ABC transporters in the MSDs (<20%), the transmembrane segments of CFTR are the most difficult to align to the Sav1866 template. This is unfortunate, since these constitute the conduction pore and are therefore critical to the protein's function as an ion channel. While these models have been validated to some degree (particularly at the interface between the NBDs and the MSDs [19]), a close investigation reveals discrepancies between their predicted pore structures and those expected from the available experimental data on CFTR function (see Results and Discussion sections for further details).

It also has been suggested that the inward-facing apo state of the typical ABC transporter may correspond to the C0 closed channel in CFTR. Mornon et al. [22] proposed such a structure based on homology modeling using as template a corrected X-ray crystal structure of the bacterial ABC lipid flippase MsbA [25]. However, this template suffered from poor resolution (5.5 Å), compounding the problem of alignment described above, and making it more difficult to determine the accuracy of their homology model. Recently, Furukawa-Hagiya et al. [26] used the high resolution, nucleotide-free, inward-facing crystal structure of mouse P-glycoprotein [27] as a template for another CFTR closed state homology model.

Here we report new homology models of the open and closed states of CFTR which address the problems described above. We used experimentally derived constraints to create a Sav1866-based model of the CFTR channel in what would be equivalent to the major open channel state (hereafter referred to as O-CFTR) that better conforms to the best available experimental predictions about the structure of the ion conduction pathway. We also created a closed state CFTR model (C0-CFTR), equivalent to the state where the NBDs are fully dissociated, using (like Furukawa-Hagiya et al. [26]) the high-resolution nucleotide-free inward-facing crystal structure of murine P-glycoprotein [27] as a template. We then performed targeted molecular dynamics (TMD) simulations to investigate the conformational changes that may underlie the gating transition in CFTR. We analyzed our trajectory for the formation and dissociation of novel amino acid side chain interactions that may help to stabilize CFTR structure, and obtained experimental validation for a novel salt bridge predicted by our simulations.

## Results

### New Homology Model of the Open CFTR Channel

**Figure 1:**
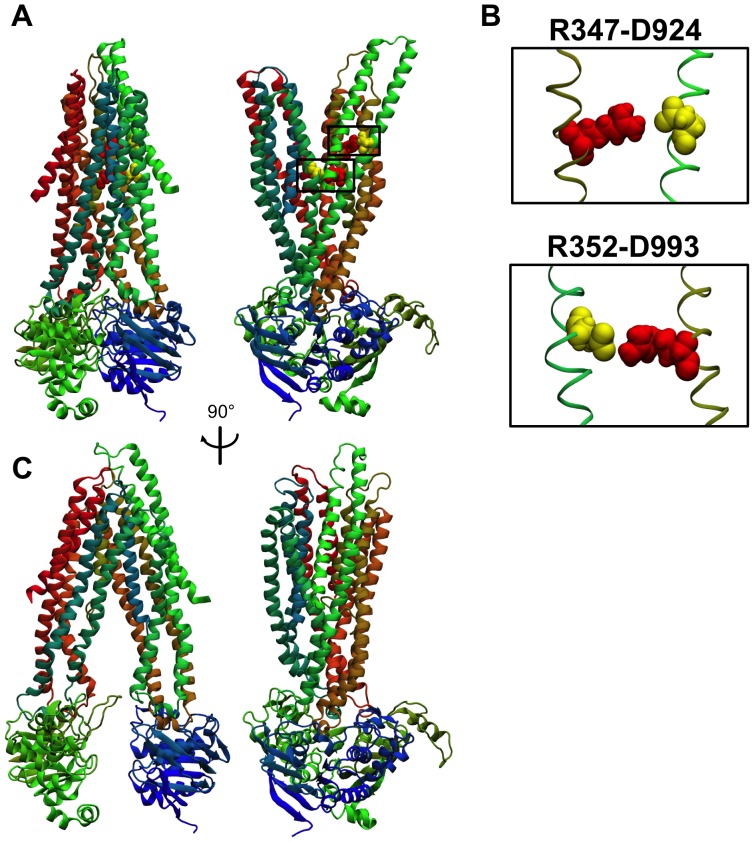
New open- and closed-state models. A: Front and side views of a ribbon representation of the O-CFTR homology model based on the Sav1866 crystal structure template. Residues are assigned colors as follows: MSD1 is colored red-orange; NBD1 green; MSD2 blue-green; and NBD2 blue. We note tight dimerization between the two NBDs, and a separation in the two “wings” of the MSDs in the transmembrane and extracellular regions. B: Close-ups of the residue pairs R347-D924 and R352-D993. The current model agrees with experimental data on salt bridge interactions between the arginine (red spheres) and aspartate (yellow spheres) residues in each of these pairs. Side chain distances between guanidinium N and carboxyl O atoms in these pairs are 4.6 Å (R347-D924) and 3.7 Å (R352-D993), respectively. C: Front and side views of a ribbon representation of the C0-CFTR model based on the P-gp crystal structure template. The same coloring scheme as in part A was followed. In this model, the NBDs are widely separated, with no separation of the “wings” in the extracellular region that constitutes a gate to ion conduction. A wide cytosolic vestibule could allow binding of large pore blockers as well as large substrates such as glutathione.

We created a homology model of the open-channel state of CFTR (without the R-domain) based on a Sav1866 template using MODELLER [28]. Due to low sequence homology (particularly in the MSDs), primary structure alone could not be used to generate a reliable alignment. Instead, a combination of secondary structure predictions, hydropathy analysis, and pore accessibility indicators were incorporated into the alignment (final alignment of the MSDs is given in **Figure S1(a)**). Soft semi-harmonic restraints were manually added between particular residues in order to ensure that established salt bridges and distance constraints derived from disulfide crosslinking experiments in CFTR double cysteine mutants were satisfied (for a list of homology modeling restraints used, see **Table S1**). Ten such models were generated and energy-minimized using a simulated annealing protocol, then ranked based on maintenance of the established R347-D924 [29] and R352-D993 [30] salt bridges, and accessibility of residues in TM6 and TM12 predicted to be pore-lining. The model that best fit these criteria (designated O-CFTR) was chosen for further study (**Figure 1A**).

**Figure 2:**
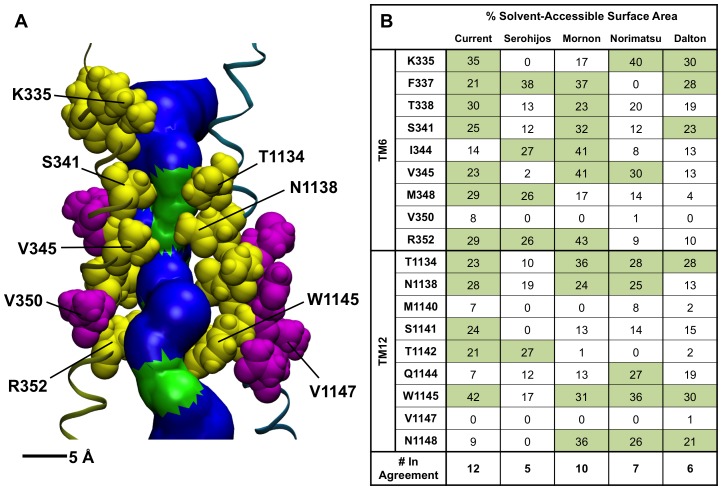
Comparison of five available open-state models. **A:** Close-up of the transmembrane pore region in O-CFTR. The predicted pore is visualized using HOLE [58] and VMD , and shown in blue (radius > 3.0 Å) and green (radius < 3.0 Å). We note the existence of an inner and an outer vestibule, separated by a narrow region flanked by the selectivity-conferring S341 residue on TM6 and T1134 on TM12. Pore-lining residues in sites that were both found to be reactive to MTS reagents in previously published experiments, irrespective of channel state and predicted by our O-CFTR model to be surface accessible are shown as yellow spheres. Residues that were expected to be pore-lining from experiment but do not appear so in our model are colored purple. **B:** A comparison of the surface accessibility of sites on TM6 [30]–[43] and TM12 [17], [31], [36], [44]–[46] predicted to be accessible from experiment in our model and others (Serohijos [19]; Mornon[20]; Norimatsu: 5 ns snapshot from [23]; Dalton [24]).

By design, the O-CFTR model contains several structural features expected to be found in the real CFTR structure. The established R352-D993 and R347-D924 salt bridge residue pairs are observed in our model to have side chains oriented toward each other with distances <5 Å between their interacting guanidinium and carboxyl groups (**Figure 1B**). In addition, a review of the surface accessibility status of predicted pore-lining residues in the critical TM6 [30]–[43] and TM12 [17], [31], [36], [44]–[46] helices (**Figure 2**) reveals that the single snapshot represented by O-CFTR has more of these residues exposed to the aqueous conduction pore than several previous open-state models, including those of Serohijos et al. [19], Mornon et al [20], Dalton et al. [24], and the 5 ns snapshot of the 30 ns molecular dynamics (MD) simulation obtained from Norimatsu et al. [23]. Of 18 residues expected to be pore-accessible based on cysteine scanning and open pore blocker experiments, 12 were predicted to be accessible (>20% surface area accessible to solvent [47]) in our model (**Figure 2B**). Decreased sensitivity to open pore blockers by mutations at V350 [43] have suggested its important role in CFTR channel structure, but none of the Sav1866-based models, including the current model, show it to be accessible from the pore. V1147 on TM12, also thought to be pore-accessible [43], [45], is similarly buried in all the models. This suggests that the effects of mutations at V350 and V1147 are indirect, or that a departure of CFTR structure from the Sav1866 template exists at the cytosolic end of the transmembrane region. Indeed, a drawback of our model is that there is no clear entrance to the transmembrane pore from the cytoplasmic end, a consequence of the alternating-access exporter function of the Sav1866 template which is shared by most existing homology models of the open CFTR channel.

Close inspection of the pore structure in the transmembrane region of our O-CFTR model reveals it to be continuously larger than 3.6 Å (the diameter of a dehydrated chloride ion [48]), indicative of an open channel (**Figure 2A**). We note the existence of a narrow region, centered upon S341 in TM6 and T1134 in TM12, which separates the transmembrane pore into an inner and outer vestibule. This pore architecture is consistent with recent predicted models [23], [24], as well as with experimental evidence suggesting the existence of a wide inner vestibule capable of binding pore-occluding blockers [49], the narrowing of the pore in the region of S341 [23], and of the functional role of S341 in anion selectivity in CFTR [32].

### New Homology Model of the Closed CFTR Channel

In addition to a Sav1866-based open state model, we also created a model of the proposed nucleotide-free closed state of CFTR using the inward-facing X-ray crystal structure of murine P-glycoprotein (P-gp) [27] as a template. Prior to generating a sequence alignment between CFTR and P-gp, we noted a discrepancy in the sizes of the extracellular loops (ECLs) in the P-gp structure and our proposed O-CFTR model. In particular, ECL1 between TM1 and TM2 in P-gp (∼41 residues long) was much larger than ECL1 in O-CFTR (∼12 residues), but similar in length to ECL4 between TM7 and TM8 in O-CFTR (∼33 residues) (**Figure S2**). Therefore, in order to facilitate modeling, like Furukawa-Hagiya et al. [26] we chose to use P-gp’s MSD1 as a template for CFTR’s MSD2 and *vice versa.* A similar procedure to that used in generating the open state model was then followed, creating a sequence alignment between CFTR and mouse P-gp and manually adjusting it to ensure a topological match between the O-CFTR model already generated and the closed state model (final alignment of the MSDs is given in **Figure S1(b)**). The final closed state homology model (C0-CFTR, **Figure 1C**) was created in MODELLER, and validated for stereochemical quality using PROCHECK [50].

As may be expected from the structure of the P-gp template, the NBDs in C0-CFTR are completely dissociated, and the MSDs tilt toward each other to form a constriction near the extracellular end of the transmembrane region. This gate, comprised of the extracellular-end residues in TM6, TM12, TM1 and TM7 along with ECL1, ECL3, ECL4, and ECL6, and perhaps with contributions from N-linked glycans (not modeled) in ECL4, provides an effective barrier to permeation, consistent with the observation that the CFTR channel is closed in the absence of NBD dimerization.

### State-Dependent Accessibility of R334C

**Figure 3:**
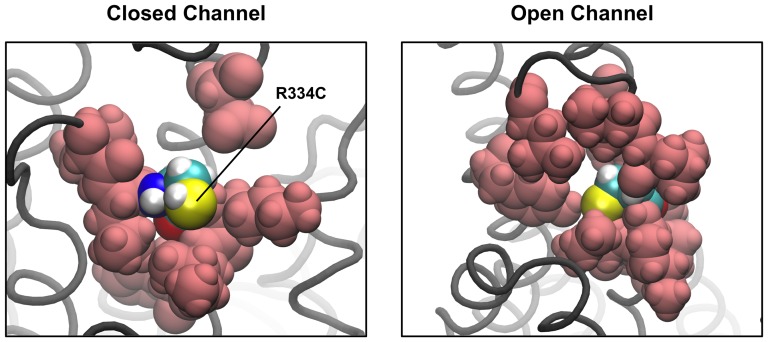
State dependent-accessibility of R334C. These images show CFTR from the extracellular side, with the mutant residue R334C represented in CPK colors, and all residues with atoms within 5 Å of R334C shown as pink spheres. R334C is largely buried by its neighboring residues in the open channel state, exposing only 3.8% of its surface area to solvent. On the other hand, in the closed channel, R334C is more exposed (32.9% accessible). This is in accordance with experimental results demonstrating the preferential closed-state accessibility of R334C [51], [52].

The arginine at position 334 on TM6 lies at the outer mouth of the pore, and has been suggested to play a role in attracting anions [33], [34]. It has been observed, however, that modification of cysteine mutants at this site (R334C) occurs only in the closed state, with the site apparently being inaccessible when the channel is open [51]–[53]. Norimatsu et al [53] have suggested that this state-dependent accessibility may be due to movement of residues neighboring R334, and a comparison of this region in our two models provides support for this hypothesis (**Figure 3**). In an open state O-CFTR model in which the arginine at position 334 was mutated to a cysteine *in silico*, R334C is buried by many of its neighboring residues in ECL3 and is found to have a fractional solvent-accessible surface area (SASA) of only 3.8%. By comparison, the C0-CFTR model presents R334C in a relatively exposed conformation, with 32.9% of its surface area accessible to solvent. This increased accessibility may translate to the higher observed rates of reaction of R334C with extracellular sulfhydryl-modifying reagents in the closed state. A similar SASA analysis of the C0-CFTR and O-CFTR models with the native arginine residue reveals that this position may exhibit similar state-dependent accessibility in wildtype CFTR (**Figure S3**).

### Targeted Molecular Dynamics Simulations

We employed TMD simulations to generate one possible molecular trajectory of a channel opening event, a prediction which was then confirmed by experiment. Our initial simulation system consisted of the C0-CFTR homology model embedded in a POPC lipid bilayer membrane patch and solvated in a 150 mM KCl solution with explicit TIP3P water molecules [54] (**Figure S4**). A biasing force was applied evenly across the whole molecule to reach the O-CFTR target structure over a 10 ns simulation, arriving at a final RMSD between the simulation structure and O-CFTR of ∼0.3 Å.

**Figure 4:**
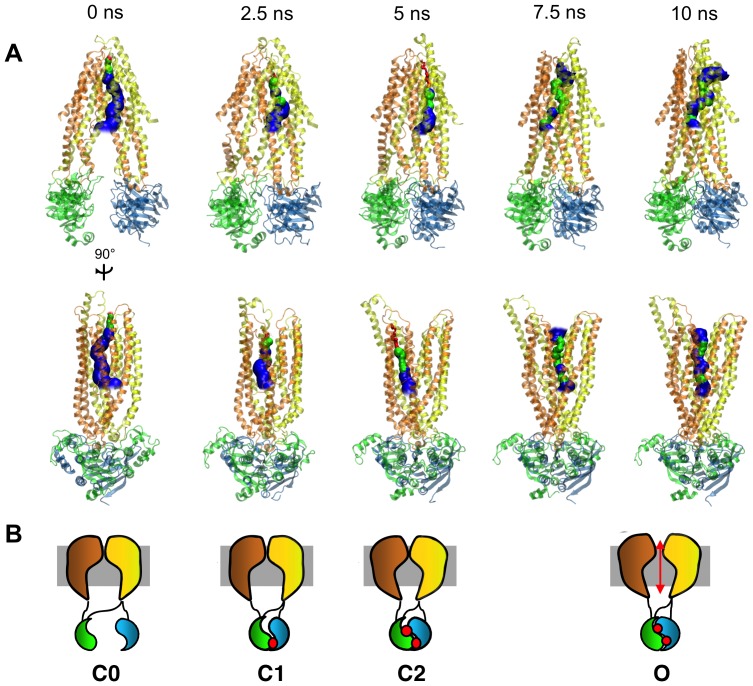
Snapshots along the simulation. **A:** Front (top row) and side (bottom row) views of snapshots taken at 0, 2.5, 5, 7.5, and 10 ns of the targeted molecular dynamics trajectory, showing the protein in ribbon representation. The predicted pore is shown in each snapshot in blue (radius > 3 Å), green (3 Å > radius > 1.8 Å), and red (radius < 1.8 Å), with red indicating a constriction too narrow to allow passage of a chloride ion. Over the first 5 ns, we observe the NBDs coming together, and increased bending in the intracellular loops (ICLs), although the transmembrane pore remains occluded. At 7.5 ns, we note that the ICLs have straightened out as the NBDs become even more tightly bound, and the conformational wave moves up to the transmembrane region where a through pore open to chloride conduction is first seen. At the end of the simulation (10 ns), tight coupling of the NBDs is complete, and the open pore is observed. **B:** Schematic representations of the fully dissociated C0, partial dimer C1, full-dimer closed transition state C2, and O open state configurations that likely correspond to the structures observed in the TMD simulation.

Snapshots of the TMD trajectory reveal a progressing “conformational wave” during the opening transition (**Figure 4**), similar to that seen in nicotinic acetylcholine receptors during their transition between closed and open states [55]. This comprises three main stages: 1) pushing together of NBDs and contraction of the inner vestibule (C0 C1 states), 2) NBDs forming a tight dimer and bending at the intracellular extensions of the TM helices that form the intracellular loops (ICLs) (C1 C2), and 3) propagation of conformational change through TM helix rotation plus translation of the two transmembrane “wings” of the molecule — comprised of TM1 + TM2 + TM9 + TM10 + TM11 + TM12 and TM7 + TM8 + TM3 + TM4 + TM5 + TM6, respectively — away from each other in the direction perpendicular to the NBD dimer interface. This NBD-initiated transition pathway is consistent with the notion that ATP binding regulates channel gating by driving conformational changes in the transmembrane pore [16].

Due to the similarity of its asymmetric nucleotide binding domains to those proposed in CFTR, a recently published crystal structure of the bacterial ABC transporter TM287/288 [56] that has nucleotide bound in the degenerate catalytic site may serve as a useful template for the C1 partial dimer state of CFTR. A comparison of the NBDs from this structure to those in our snapshots confirms that the 2.5 ns snapshot of our TMD trajectory most closely resembles the purported partial dimer closed state of TM287/288 (**Figure S5**).

### Tracking Conformational Changes

**Figure 5:**
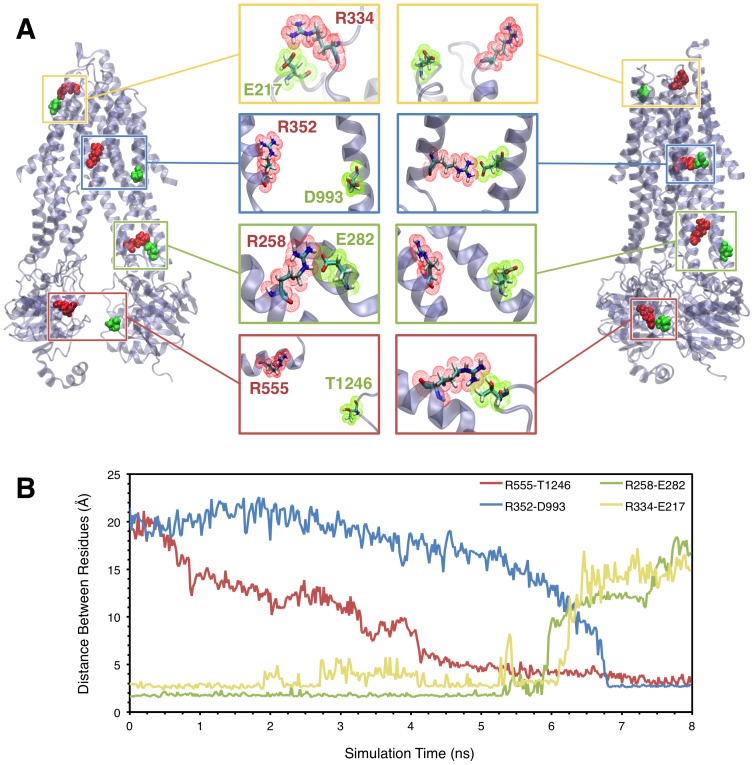
Tracking the conformational changes in CFTR channel opening by following particular side-chain interactions. **A** shows close-ups of the R352-D993 salt bridge [30] and the R555-T1246 inter-NBD hydrogen bond [16], which exist only in the open state, and the proposed novel R258-E282 and R334-E217 salt bridges which exist only in the closed state. Tracking the formation and breaking of these interactions over the course of the simulation (**B**) shows that the NBDs form a stable dimer at around 4 ns. The conformational wave then proceeds upwards through the ICLs, where the R258-E282 salt bridge is broken at ∼6 ns, and then to the transmembrane region where the R352-D993 salt bridge is formed nearly 7 ns after the beginning of the simulation. The point at which the R334-E217 salt bridge that stabilizes the closed state breaks is also indicated (∼6.3 ns). Distances between residues in part (B) are measured between interacting side chain heavy atoms, specifically the guanidinium N of arginines, carboxyl O of E282 and D993, and hydroxyl O of T1246.

In order to track the progression of the conformational wave as it travels from the NBDs through the ICLs to the transmembrane region, we charted the formation and breaking of the residue pair interactions shown in **Figure 5A** over the course of the simulation. In the NBDs, we tracked the distance between the side chains of residues R555 (NBD1) and T1246 (NBD2), which have been suggested to participate in a hydrogen bond interaction upon NBD dimerization [16]. In the TM helices, the formation of the R352-D993 [30], [57] salt bridge was followed. In the ICLs, we discovered a novel salt bridge interaction between residues R258 and E282 in our simulations that exists in the closed state but is broken in the transition to the open channel as the passing conformational wave causes these residues to move apart.

Tracking these interactions over the course of the 10 ns simulation confirms the overall direction in which the transition progresses (**Figure 5B** **)**. The distance between R555 and T1246 decreases monotonically over the first 4 ns of the simulation as the NBDs were pushed together, and is then locked with the formation of a stable hydrogen bond at ∼5 ns. The breaking of the closed-state R258-E282 salt bridge follows at ∼6 ns, as the intracellular extensions of TM4 and TM5 slide past each other with the contraction of the cytoplasmic vestibule. The conformational wave then leads to rotations in TM6 and TM9, bringing residues R352 and D993 closer together until a stable salt-bridge is formed between them after ∼7 ns.

**Figure 6:**
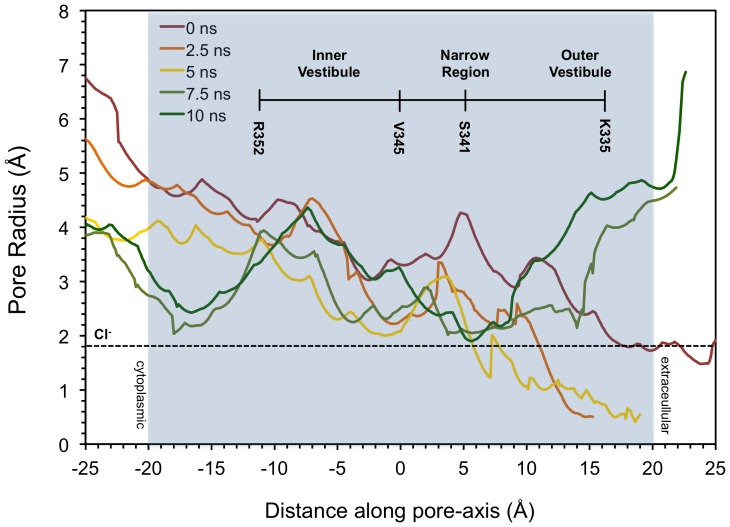
Tracking the pore radius profile over the course of the simulation. Pore radius at different points along the transmembrane pore axis for the 0, 2.5, 5, 7.5, and 10 ns snapshots of the TMD trajectory using HOLE [58]. The first 5 ns of the simulation are characterized by a reduction in the transmembrane (shaded region) pore radius as the NBDs are pushed together, resulting in a pore that is too constricted for chloride conduction at the extracellular end. After 7.5 ns, the conformational changes propagate to the TM region, resulting in an expansion in the transmembrane pore that is complete by the 10 ns snapshot.

The pore radius profile at different points of the simulation provides another means of tracking the transition (**Figure 6**). Snapshots of the trajectory at 0, 2.5, 5, 7.5, and 10 ns were saved and the pore radius along the pore axis in each was determined using the program HOLE [58]. Over the first 5 ns, we noted a contraction of the inner vestibule, presumably as a result of the cytosolic domains being pushed together. During this period, the conduction pore radius is never larger than a chloride ion throughout its length, demonstrating that the channel is indeed closed when the NBDs are dissociated or in the early stages of dimer formation in our simulation. Subsequently, the formation of a tight NBD dimer results in conformational changes in the TM region leading to its expansion. A through-pore that is continuously larger than a chloride ion develops at 7.5 ns and is maintained through the end of the simulation, and is characterized by an inner vestibule and a large outer vestibule separated by a “narrow region” centered around S341 in TM6. Such a pore profile is in agreement with experimental data suggesting the existence of a narrow region and anion selectivity filter around S341 [32], [43].

### Side-chain Interactions

As the channel opening transition sweeps through the structure, the local microenvironment around each residue could change as a result of backbone translation and rotation, exposing them to interactions with various neighboring residues over the course of the transition. We analyzed all 104 basic arginine and lysine residues in our models for close contacts (≤5 Å separation between side chains) with neighboring negatively charged (aspartate or glutamate) and hydroxyl-containing (serine, threonine, tyrosine) amino acids for salt bridge and hydrogen-bonding interactions, and classified them as being closed-state (existing primarily over the first half of the channel opening TMD simulation), open-state (existing primarily in the latter half), or persistent interactions. Beside the previously established R347-D924, R352-D993, and R555-T1246 interactions [10], [16], [29], [30], the results shown in **Table S2** predict the existence of 89 additional amino acid interactions that have not previously been reported. These interactions involve residues that have been linked to 86 different CF-causing missense and deletion mutations [59]. We hypothesize that these interactions may be vital either to stabilizing a particular CFTR state or to the overall structural integrity of the protein.

### Experimental Support for the Models

**Figure 7:**
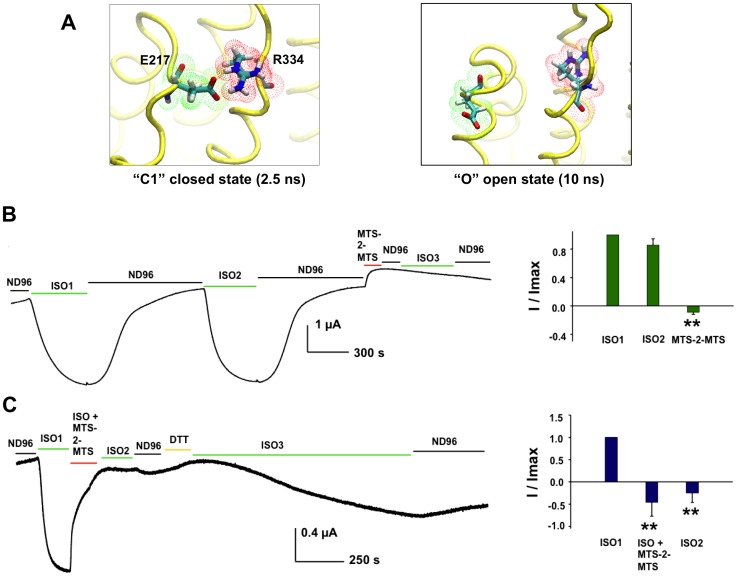
Crosslinking R334C to E217C locks CFTR channels into the closed state. **A:** TMD simulations predicted a salt bridge interaction between residues R334 and E217 that persists throughout the initial C0 inward-facing state, C1 partial dimer closed state, and C2 closed transition states over the first 5 ns of the simulation. This salt bridge was predicted to break during channel opening as the ECLs move apart during the last 5 ns of the simulation. **B:** Representative trace (*left*) and summary data (*right*) for macroscopic currents measured from R334C/E217C-CFTR with addition of the crosslinker MTS-2-MTS in the absence of isoproterenol (ISO), used to activate channels. ND96  =  control bath solution. **C:** Representative trace and summary data for currents measured from R334C/E217C-CFTR with the crosslinker MTS-2-MTS added in the continuing presence of isoproterenol. DTT  =  dithiotreitol reducing agent. Current levels in the summary data are given relative to control conditions prior to first exposure to ISO, and normalized to maximal current in response to ISO1. **  =  p<0.001 compared to ISO1 in n  =  4 experiments each.

One of the intraprotein interactions suggested by our modeling effort, which has not been indicated in previous models, is the R334-E217 salt bridge (**Figure 5A**). This salt bridge is seen in our simulations to be stable throughout the proposed C0, C1, and C2 closed states, finally breaking after approximately 6.3 ns of the simulation have elapsed — following the formation of the tight NBD dimer and changes in interactions in the ICLs — during the final transition to the open channel O state (**Figure 5B**). To provide experimental confirmation of the closed and open structures, and the transitions between the two, we asked whether cysteines engineered at these two positions in the double-mutant, R334C/E217C-CFTR, could be functionally crosslinked. We expressed the double mutant channels in *Xenopus* oocytes, and measured macroscopic currents (see Methods). The traces in **Figure 7** show that R334C/E217C-CFTR can repeatedly be activated by stimulation of the co-expressed beta2-adrenergic receptor using isoproterenol, without substantial decrement in peak current prior to exposure to the crosslinker MTS-2-MTS. When the same cell was exposed to MTS-2-MTS in the absence of isoproterenol (**Figure 7B**), when most of the channels should be closed, subsequent exposure to isoproterenol failed to activate CFTR channels to the same degree as prior to MTS-2-MTS; these results are consistent with the notion that R334C and E217C are positioned very near each other in the channel closed state. In the lower trace (**Figure 7C**), the cell was exposed to MTS-2-MTS in the continuing presence of isoproterenol, which resulted in a rapid decrease in current; after washout of isoproterenol and crosslinker, channels could not be reactivated by a second exposure to isoproterenol alone. When fully activated under physiological conditions, CFTR channels are only open ∼40% of the time [60]. The loss of macroscopic current in this experiment, then, represents trapping of channels in the closed state by the MTS-2-MTS crosslinker. Subsequent exposure to DTT to break the disulfide bond(s) enabled activation upon re-application of isoproterenol (**Figure 7C**). In control experiments, exposure to MTS-2-MTS did not have similar effects on the single mutants R334C-CFTR and E217C-CFTR, with respect to the ability to re-open channels after MTS-2-MTS exposure (**Figure S6**). Similarly, exposure of the double mutant to monofunctional MTS reagents did not impact the ability to activate CFTR channels upon subsequent reapplication of isoproterenol (**Figure S7**). Inspection of the C0- and O-CFTR models indicates that the side chains of R334C and E217C are, indeed, close enough to be crosslinked by MTS-2-MTS only in the closed state (**Figure S8**). The results in **Figure 7** are consistent with the notion that R334 and E217 approach each other closely in the closed state of CFTR and that this salt bridge breaks as the channel opens, as predicted by our TMD simulations.

## Discussion

### New Homology Models

Understanding structure-function relationships in CFTR will aid in the development of therapeutic treatments for the underlying cause of CF. While a static molecular model may provide a framework for drug development, in reality, the CFTR pore surely occupies an ensemble of conformers — rather than a single frozen state. The dynamics and ensemble characteristics of various homology models have previously been studied using free molecular dynamics simulations rather than TMD [21], [23], [24], [26]. Data from experimental studies, in addition, provide insight about functional states (also reflecting ensembles of conformers) and interactions that are more likely than others. Our aim in this study was to create a single *consensus model* that best incorporates much of the available experimental information about the CFTR open channel structure. Previous models proposed by Serohijos et al. [19], Alexander et al. [21] and Norimatsu et al. [23] did not include the established salt bridge between residues R347 and D924 [29], and the R352-D993 [30] salt bridge interaction did not exist in the open state model of Mornon et al. [20]. Our Sav1866-based O-CFTR model correctly accommodates all of this structural information, and, like the models from Dalton [24] and Norimatsu [23] and colleagues, contains a transmembrane pore region depicting inner and outer vestibules separated by a narrow region near the selectivity-conferring residues S341 and T338 [23], [32]. Our C0-CFTR closed channel model uses a mouse P-gp template reported at much higher resolution than the MsbA structure previously used by Mornon et al. [22]; our model also included a swap of the N- and C-terminal to improve the alignment (see Methods) similar to the model recently published by Furukawa-Hagiya and coworkers [26].

Although CFTR is thought to cycle between a closed state with partially-dimerized NBDs (C1) and a tight-dimer open state (O) during its action as a chloride channel, it is also known to transport large substrates such as the glutathione antioxidant tripeptide (GSH) [61]. Given the disparity in size between the chloride ion and GSH, transport of GSH may require the much larger inner vestibule provided by the fully NBD-dissociated state represented in our C0-CFTR model. As shown in **Figure 6**, the ion channel pore/putative substrate binding pocket exhibits a fairly wide diameter to nearly its external end in the 0 ns model, which is somewhat collapsed during the first 5 ns of the simulation.

While some experimental data, such as the salt bridge interactions and accessibility of pore-lining residues, were built into our models as constraints, we also observe that the models accurately predict results that were not explicitly controlled for during model generation. For example, cysteine mutants at residue R334 in the outer mouth of the pore [52], [53] have been demonstrated to be more reactive to MTS reagents in the closed channel state than in the open channel. This residue is more exposed in our C0-CFTR model than in our O-CFTR model. These findings give us confidence in the predictive power of these models and in their ability to explain and interpret functional data in terms of specific structural changes in the CFTR molecule. It is our hope that these improved models of CFTR will serve as a useful visualization tool for experimenters in designing and interpreting results from structure-function studies, and as an accurate reference state from which to begin simulation studies of CFTR.

### Targeted Molecular Dynamics Simulations

Recent studies have employed MD simulations to sample the conformational space around particular CFTR homology models [21], [23], [24], [26]. Simulating large-scale conformational transitions using conventional MD, however, is unfeasible in typical timescales. In a recent study by Furukawa-Hagiya et al. [26], a free MD simulation starting with a homology model of the CFTR closed state with MgATP bound in the NBDs did not reveal any significant widening of the transmembrane pore over 100 ns. In the present study, we used targeted molecular dynamics to generate a trajectory of one possible transition pathway between C0-CFTR and O-CFTR. Although the use of a biasing force in this technique prevents definite prediction of the true transition pathway, TMD has been successfully employed to study several other ABC proteins [62], [63], and it can provide valuable information about events that may take place during the transition, including large-scale motions of backbone structures, and the changing interaction partners and surface accessibility of particular residues as the transition progresses.

It should be noted that, while the pattern of changes in interactions exhibit a general trend from the NBDs upward, the vertical position along the pore axis of a particular interacting pair in our simulation is not an absolute determinant of the order in which it changes. Thus, while the closed-state salt bridge pair R334-E217 is located in the extracellular loops — further away from the NBDs than any of the other pairs shown in **Figure 5** — it appears to break at 6.3 ns, before transmembrane R352-D993 salt bridge is formed, indicating that at least partial freeing of the ECLs may be required for the helical rotations that lead to the transmembrane pore opening.

In our simulations the most significant change during the initial stages of the transition from C0 (0 ns) involves the translation of the NBDs toward each other and the concomitant contraction of the cytoplasmic vestibule below R352, as suggested by **Figure 6**. Such an NBD-initiated transition is consistent with the long-standing hypothesis that ATP binding at the NBDs initiates channel opening in CFTR [16]. After 2.5 ns, the NBDs come into close approach, but the R555-T1246 inter-NBD hydrogen bond has not yet formed, and the channel is closed to ion conduction (**Figures 4** **,** **5**). Of the trajectory snapshots analyzed, the NBDs of the 2.5 ns snapshot have the lowest RMSD when superimposed onto the NBDs of the recently crystallized TM287/288 bacterial ABC transporter [56] which, like CFTR, has asymmetric NBDs containing one degenerate nucleotide binding site. This suggests that the 2.5 ns snapshot may approximate the C1 partial dimer state that CFTR primarily occupies when closed to ion conduction. Halfway through the trajectory, we observe a state where the NBDs are close enough for a hydrogen bond between R555 and T1246 to form, but the pore radius in the transmembrane region still remains too constricted to allow the passage of a chloride ion. This configuration may represent the ATP-bound full dimer closed channel transition state that CFTR is thought to adopt during its gating cycle (C2) [16]. A thermodynamic analysis by Csanády et al. [64] suggested that this transition state contains a high degree of conformational strain, and analysis of our simulation trajectory reveals kinks in the intracellular loop regions in this state that may contribute to this. Lending further support to the 5 ns structure as a model for the C2 closed transition state, we note that known open-state interactions such as the R352-D993 salt bridge have not yet occurred at this stage (**Figure 5B**). In the latter half of the simulation, there is an expansion of the transmembrane pore region (**Figure 6**), culminating in a through-pore with radius continuously greater than that of a chloride ion (**Figure 4**). As the O-CFTR target state is approached, the structure contains inner and outer vestibules separated by a (possibly selectivity-conferring) narrow region. We note that this narrow, open region persists during the entire transition between the C1 and O states in our simulations, despite a widening of the pore in the outer vestibule. The stability of this region was demonstrated by Liu et al. [65] who showed that the constriction in this region to extracellularly applied reagents remains stable over temperatures from 22°C to 37°C, despite major conformational changes in the outer vestibule.

As the only ABC protein known to have channel-like properties, insights into CFTR structure are difficult to extract based solely on studies of the crystal structures of one state or another in other ABC transporters. Our simulations provide insights into the nature of the C1 partial dimer and C2 closed transition state and the large-scale motions involved in the transition between these various states.

### Novel Predicted Side-Chain Interactions

One of the most promising aspects of our simulations is the ability to track the formation and dissociation of specific residue pair interactions as the backbone structures are transformed between the various channel states, and we report here possible contacts for all positively charged (arginine and lysine) residues over the course of the transition (**Table S2**). These predictions will guide experiments to further define the structure of this unique and unusual protein, and, in this study, a novel salt bridge between R334 and E217 predicted to break during channel opening was confirmed experimentally using functional crosslinking (**Figure 7**); this result also helps explain the heretofore unclear role of R334 in CFTR function [34], [51], [52]. Notably, many of the amino acids that participate in these predicted interactions are also involved in CF disease-associated mutations. These predictions should offer insight into the structural defects that lead to CFTR dysfunction in these mutants, and, ultimately, into mechanisms that could enable their correction.

## Materials and Methods

### Homology Modeling of the Open State

A homology model of the open state of CFTR was created using the crystal structure of Sav1866 (PDB: 2HYD) [18] as a template. First, a sequence alignment between CFTR and Sav1866 in the program AlignMe [66] was generated by combining sequence homology (using the BLOSUM62 substitution matrix), a sliding-window average hydrophobicity profile (using the KD hydrophobicity scale [67]), PSIPRED predictions of secondary structure [68], and OCTOPUS transmembrane topology predictions [69]. In addition, the correct alignment of buried and exposed residues in TM6 and TM12 was enforced by adding a custom measure of similarity to the AlignMe input. A profile of percentage solvent accessible surface areas was generated for each residue in the Sav1866 template in TM6 and TM12 using VMD [70], and this was aligned to a counterpart profile on the CFTR sequence containing predictions of pore-accessibility of residues in TM6 and TM12 based on published experimental data (residues were assigned a score of 100 if thought to be accessible from the pore, 0 if buried). The program then used this information to align residues in the CFTR sequence thought to be pore-accessible to sites on the Sav1866 template with high solvent-accessible surface areas. The region of the CFTR sequence containing the R-domain (residues 650 through 856) was omitted from all of the modeling in this study, since no analogous structure exists in the ABC templates, and it is in fact thought to be largely unstructured [15]. The Sav1866 template structure was crystallized with ADP at both NBD binding sites, but these were removed in our homology models. Homology modeling was carried out in Modeller v. 9.10 [28], with the addition of minimum distance restraints between the salt bridge pairs R347-D924 and R352-D993, as well as restraints between residues thought to be cross-linkable (for a complete list of distance restraints used, see **Table S1**). Ten models were generated, and the best one — based on observed maintenance of the known salt bridges and solvent accessibility of predicted pore-lining residues in TM6 and TM12 – was selected for further study (O-CFTR). Stereochemical quality of the final model was validated using PROCHECK [50]. The radius profile of the ion conduction pore was analyzed using the program HOLE [58] along the axis normal to the lipid bilayer. **Figure S9** presents a Ramachandran plot for the O-CFTR model.

### Homology Modeling of the Closed State

A similar procedure to that described above was used to generate a sequence alignment between CFTR and the closed state template, murine P-glycoprotein (PDB:3G5U) [27], using sequence, hydrophobicity and predicted secondary structure information. An initial comparison of the lengths of the extracellular loops revealed similarities between MSD1 of CFTR and MSD2 of P-gp and *vice versa* (**Figure S2**), and these domains were thus switched during the modeling procedure. Given the low sequence homology between P-gp and CFTR in the MSDs, it is not possible to determine whether this domain switching has evolutionary significance, although the assertion that full-transporters such as CFTR and P-gp may have been independently assembled from ancestral half-transporter genes [10], [71] does not preclude this possibility.

Unlike the open-state model, there is a dearth of structural information that could be incorporated in our modeling of the closed state. Anchors for the alignment were, however, provided by the expectation that gross reorganization of the transmembrane topology would not occur in the transition from the closed state to the open state: residues embedded in the transmembrane region in the creation of the O-CFTR model were expected to persist in the membrane in the closed state model. Using predictions of the position of the transmembrane region from the Orientation of Proteins in Membranes (OPM) database [72], the generated sequence alignment was checked and manual adjustments were made where needed in order to ensure a topological match between the generated closed state model and the previously generated O-CFTR model; residues predicted to be in the transmembrane region in the O-CFTR model were aligned to corresponding transmembrane residues in the P-gp template. No additional restraints were used during modeling in Modeller 9.10 [28]. PROCHECK [50] was used to check stereochemical quality of the generated C0-CFTR model which revealed 93% of the residues to be in the most-favored regions, 7% in allowed regions, and none in disallowed regions.

### Targeted Molecular Dynamics

In order to carry out a realistic simulation of a channel opening event, the closed state-protein model, C0-CFTR, was first embedded in a membrane environment and then solvated and ionized using VMD 1.9.1 [70]. A 120 Å×120 Å membrane patch consisting of 343 POPC residues was generated, into which the protein was inserted based on the calculated transmembrane position of the P-gp template recorded in the Orientation of Proteins in Membranes database [72]. The system was then solvated and neutralized by the addition of TIP3P water molecules [54] (a 3-site water model for which the CHARMM27 force field has been optimized), 146 K^+^ atoms and 155 Cl^−^ atoms (ionic strength 150 mM) to form a 120 Å×120 Å×160 Å simulation box (**Figure S4**). The full system comprised 218,356 atoms. NAMD 2.8 [73] was used for simulating this system, using the CHARMM27 force field [74] in a Langevin temperature and pressure controlled (*NPT* @ 300K) ensemble and periodic boundary conditions with particle-mesh Ewald electrostatics. Following energy minimization to remove van der Waals clashes within the system, the lipid membrane tails were first allowed to “melt” together to remove gaps in the generated patch, while holding all other components of the system fixed. This was followed by a 10 ns molecular dynamics (MD) equilibration of the entire system with only the protein held restrained. Targeted molecular dynamics (TMD) simulations were then carried out through the addition of a TMD potential to the force field of the backbone α-carbon atoms, of the form: 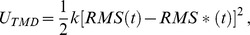



where *RMS*(*t*) was the instantaneous best fit RMS distance of the coordinates at time *t* from the target coordinates (the O-CFTR model), and RMS*(*t*) evolved linearly from the initial RMSD to zero over the course of the 10 ns simulation, thus having the effect of reducing the RMSD between the protein and the target as the simulation progressed. The TMD force constant, *k*, was set to 5 kcal/(mol Å^2^). Snapshots of the simulation trajectory taken at 0 ns, 2.5 ns, 5 ns, 7.5 ns, and 10 ns were saved and their pores were analyzed using the program HOLE [58] along the axis normal to the lipid bilayer. The PDB files for each of these snapshots can be found at: http://harvey.gatech.edu/cftr/PLoSONE_2013/.

### Electrophysiology Experiments

Experimental approaches were identical to those described in detail previously [30], so are given here in brief form. Stage V-VI oocytes were isolated from *Xenopus laevis*, under a protocol approved by the IACUC at Emory University, and injected with cRNA encoding wildtype human CFTR or mutants bearing cysteines engineered at position 334 and/or 217 on the wildtype background. Macroscopic currents were measured using an Axon Axoclamp 900A amplifier (Molecular Devices, Sunnyvale, CA) at a holding potential of –60 mV, and were activated upon stimulation of the co-expressed human beta2-adrenergic receptor by exposure to 10 µM isoproterenol added to the bath solution (ND96, containing in mM 96 NaCl, 2 KCl, 1 MgCl_2_, and 5 HEPES, pH 7.5). Cells were exposed to 1 mM 1,2-ethanediyl bismethanethiosulfonate (MTS-2-MTS; Toronto Research Chemicals, CANADA) in the presence or absence of isoproterenol.

## Supporting Information

Figure S1 **Template Sequence Alignments.** Sequence alignments of the membrane-spanning domains of human CFTR with (A) Sav1866 and (B) mouse P-glycoprotein indicating the positions of TM helices. Note that the P-gp MSD2 was aligned with CFTR MSD1 and vice versa.
(TIF)

Figure S2 **Possible topological swap between CFTR and P-gp.** Comparison of the topologies of the O-CFTR model (top) and P-gp (bottom) that demonstrate the discrepancy in the size of the extracellular loops between the two structures. In order to facilitate modeling, MSD1 of P-gp was used as a template for MSD2 of CFTR, and vice versa.
(TIF)

Figure S3 **State-dependent accessibility of R334.** These images show CFTR from the extracellular side, with the residue R334 represented by spheres in CPK coloring, and all residues with atoms within 5 Å of R334 shown as pink spheres. R334 is largely buried by its neighboring residues in the open channel state, exposing only 7.6% of its surface area to solvent. On the other hand, in the closed channel, R334 is more exposed (57.3% accessible).
(TIF)

Figure S4 **Setting up the simulation.** A snapshot of the unit simulation box, showing the C0-CFTR protein model (orange ribbon) embedded in an equilibrated POPC lipid membrane patch (spheres) and enclosed in a water box. Potassium and chloride ions (not shown) are included throughout the solvent.
(TIF)

Figure S5 **Comparison of NBDs from trajectory structures and TM287/288.** PyMOL (Schrödinger, LLC) was used to align, superimpose, and calculate the RMSD between NBDs from the 0 ns, 2.5 ns, 5 ns, 7.5 ns and 10 ns snapshots of the CFTR TMD trajectory with the NBDs from the asymmetric crystal structure of TM287/288 [5]. (A) Image showing superimposed NBDs from the 2.5 ns snapshot (green) and TM287/288 (red). (B) Calculated RMSDs between the NBDs from various trajectory structures and TM287/288. The 2.5 ns snapshot structure most closely resembles the TM287/288 partial dimer structure.
(TIF)

Figure S6 **Effects of 1 mM MTS2-2MTS on R334C-CFTR and E217C-CFTR channels.** Representative traces (left) and summary data (right) for macroscopic currents measured from R334C- (A) and E217C-CFTR (B) by two-electrode voltage clamp. Channels were activated by exposure of the oocyte to isoproterenol (ISO). ND96  =  control bath solution. At this concentration, MTS-2-MTS acts as a pore-blocker, reducing the current levels of both mutants. MTS-2-MTS also appeared to covalently decrease macroscopic current at R334C-CFTR, but not E217C-CFTR, perhaps due to alteration of charge or side-chain volume. However, the bifunctional MTS reagent was not capable of covalently locking closed CFTR channels bearing a single cysteine, unlike its effect on channels bearing cysteines at both positions (Figure 7). Current levels in the summary data are given relative to control conditions prior to first exposure to ISO, and are normalized to maximal current in the response to ISO1 (Imax). *, *p*<0.05 compared to ISO1. *n*  =  3 for both mutants.
(TIF)

Figure S7 **Effects of MTSET (ET+) and MTSES (ES-) on R334C/E217C-CFTR channels.** Representative traces (left) and summary data (right) for macroscopic currents measured from R334C/E217C-CFTR by two-electrode voltage clamp with addition of the 1 mM monofunctional MTS reagents MTSET^+^ (ET^+^) or MTSES^−^ (ES^−^) in the presence of isoproterenol (ISO). ND96  =  control bath solution. Both ET^+^ and ES^−^ covalently bound to R334C/E217C-CFTR. MTSET^+^ led to an increase in current, part of which decayed upon washout of reagent and thus was not covalent. MTSES^−^ led to a decrease in current, all of which appeared to be covalent. Importantly, neither MTSET^+^ nor MTSES^−^ inhibited the subsequent activation by ISO. Current changes in response to ISO are given relative to control conditions prior to first exposure to ISO1 (I_ISO1_), and current changes by covalent modification by MTS reagents are given relative to the maximal current in response to ISO1(I_MTS_). Covalent change by MTS =  I_MTS_/I_ISO1_. n  =  4 experiments each.
(TIF)

Figure S8 **Distances between residues in R334C-E217C Double Mutant.** PyMOL (Schrödinger, LLC) was used to mutate residues R334 and E217 to cysteines in the 2.5 ns (C1 partial dimer closed) and 10 ns (O open) snapshots. The distance between the side-chain sulfhydryl groups in the C1 state (2.5 ns) was found to be 5.3 Å, and in the O state (10 ns) snapshot it was 13.7 Å.
(TIF)

Figure S9 **Ramachandran plot of the O-CFTR model.** Analysis using PROCHECK [6] reveals 94.6% (910 of 962) non-glycine and non-proline residues in the most favorable "core" regions of the Ramachandran plot (colored red). Of the remainder, 4.6% (44 residues) were found in "additional allowed" regions (orange), and four residues (labeled) were in "generously allowed regions". No residues in O-CFTR were found in disallowed regions of the Ramachandran plot. Glycine residues are plotted as triangles, and all others as squares.
(TIF)

Table S1 **Homology modeling distance restraints.**
(DOCX)

Table S2 **Side-chain interactions.**
(DOCX)

References S1 **List of references cited in other Supporting Information.**
(DOCX)

## References

[1] Dean M, Hamon Y, Chimini G (2001) The human ATP-binding cassette (ABC) transporter superfamily. *J Lipid Res* 42: 1007–1017. 11441126

[2] Riordan JR, Rommens JM, Kerem B, Alon N, Rozmahel R, et al (1989) Identification of the cystic fibrosis gene: cloning and characterization of complementary DNA. *Science* 245: 1066–1073. 2475911 10.1126/science.2475911

[3] Quinton PM (1990) Cystic fibrosis: a disease in electrolyte transport. *FASEB J* 4: 2709–2717. 2197151 10.1096/fasebj.4.10.2197151

[4] Anderson M, Gregory R, Thompson S, Souza D, Paul S, et al (1991) Demonstration that CFTR is a chloride channel by alteration of its anion selectivity. *Science* 253: 202–205 doi:10.1126/science.1712984 1712984 10.1126/science.1712984

[5] Pilewski J, Frizzell R (1999) Role of CFTR in airway disease. *Physiological Reviews* 79: S215–S255. 9922383 10.1152/physrev.1999.79.1.S215

[6] Field M, Semrad C (1993) Toxigenic diarrheas, congenital diarrheas, and cystic fibrosis: disorders of intestinal ion transport. *Annu Rev Physiol* 55: 631–655. 7682048 10.1146/annurev.ph.55.030193.003215

[7] Higgins CF (1995) The ABC of channel regulation. *Cell* 82: 693–696. 7671298 10.1016/0092-8674(95)90465-4

[8] Higgins CF, Linton KJ (2004) The ATP switch model for ABC transporters. *Nat Struct Mol Biol* 11: 918–926 doi:10.1038/nsmb836 15452563 10.1038/nsmb836

[9] Basso C, Vergani P, Nairn AC, Gadsby DC (2003) Prolonged nonhydrolytic interaction of nucleotide with CFTR's NH_2_-terminal nucleotide binding domain and its role in channel gating. *J Gen Physiol* 122: 333–348 doi:10.1085/jgp.200308798 12939393 10.1085/jgp.200308798 PMC2234483

[10] Jordan IK, Kota KC, Cui G, Thompson CH, McCarty NA (2008) Evolutionary and functional divergence between the cystic fibrosis transmembrane conductance regulator and related ATP-binding cassette transporters. *Proc Natl Acad Sci U S A* 105: 18865–18870 doi:10.1073/pnas.0806306105 19020075 10.1073/pnas.0806306105 PMC2585040

[11] Lewis HA, Buchanan SG, Burley SK, Conners K, Dickey M, et al (2003) Structure of nucleotide-binding domain 1 of the cystic fibrosis transmembrane conductance regulator. *EMBO J* 23: 282–293 doi:10.1038/sj.emboj.7600040 14685259 10.1038/sj.emboj.7600040 PMC1271750

[12] Lewis HA, Zhao X, Wang C, Sauder JM, Rooney I, et al (2005) Impact of the deltaF508 mutation in first nucleotide-binding domain of human cystic fibrosis transmembrane conductance regulator on domain folding and structure. *J Biol Chem* 280: 1346–1353 doi:10.1074/jbc.M410968200 15528182 10.1074/jbc.M410968200

[13] Lewis HA, Wang C, Zhao X, Hamuro Y, Conners K, et al (2010) Structure and dynamics of NBD1 from CFTR characterized using crystallography and hydrogen/deuterium exchange mass spectrometry. *J Mol Biol* 396: 406–430 doi:10.1016/j.jmb.2009.11.051 19944699 10.1016/j.jmb.2009.11.051

[14] Rosenberg MF, O'Ryan LP, Hughes G, Zhao Z, Aleksandrov LA, et al (2011) The cystic fibrosis transmembrane conductance regulator (CFTR): three-dimensional structure and localization of a channel gate. *J Biol Chem* 286: 42647–42654 doi:10.1074/jbc.M111.292268 21931164 10.1074/jbc.M111.292268 PMC3234965

[15] Baker JMR, Hudson RP, Kanelis V, Choy W-Y, Thibodeau PH, et al (2007) CFTR regulatory region interacts with NBD1 predominantly via multiple transient helices. *Nat Struct Mol Biol* 14: 738–745 doi:10.1038/nsmb1278 17660831 10.1038/nsmb1278 PMC3943242

[16] Vergani P, Lockless SW, Nairn AC, Gadsby DC (2005) CFTR channel opening by ATP-driven tight dimerization of its nucleotide-binding domains. *Nature* 433: 876–880 doi:10.1038/nature03313 15729345 10.1038/nature03313 PMC2756053

[17] Bai Y, Li M, Hwang T-C (2011) Structural basis for the channel function of a degraded ABC transporter, CFTR (ABCC7). *J Gen Physiol* 138: 495–507 doi:10.1085/jgp.201110705 22042986 10.1085/jgp.201110705 PMC3206304

[18] Dawson RJP, Locher KP (2006) Structure of a bacterial multidrug ABC transporter. *Nature* 443: 180–185 doi:10.1038/nature05155 16943773 10.1038/nature05155

[19] Serohijos AWR, Hegedus T, Aleksandrov AA, He L, Cui L, et al (2008) Phenylalanine-508 mediates a cytoplasmic-membrane domain contact in the CFTR 3D structure crucial to assembly and channel function. *Proc Natl Acad Sci U S A* 105: 3256–3261 doi:10.1073/pnas.0800254105 18305154 10.1073/pnas.0800254105 PMC2265173

[20] Mornon J-P, Lehn P, Callebaut I (2008) Atomic model of human cystic fibrosis transmembrane conductance regulator: membrane-spanning domains and coupling interfaces. *Cell Mol Life Sci* 65: 2594–2612 doi:10.1007/s00018-008-8249-1 18597042 10.1007/s00018-008-8249-1 PMC11131860

[21] Alexander CS, Ivetac A, Liu X, Norimatsu Y, Serrano JR, et al (2009) Cystic fibrosis transmembrane conductance regulator: using differential reactivity toward channel-permeant and channel-impermeant thiol-reactive probes to test a molecular model for the pore. *Biochemistry* 48: 10078–10088 doi:10.1021/bi901314c 19754156 10.1021/bi901314c PMC2765204

[22] Mornon J-P, Lehn P, Callebaut I (2009) Molecular models of the open and closed states of the whole human CFTR protein. *Cell Mol Life Sci* 66: 3469–3486 doi:10.1007/s00018-009-0133-0 19707853 10.1007/s00018-009-0133-0 PMC11115851

[23] Norimatsu Y, Ivetac A, Alexander CS, Kirkham J, O'Donnell N, et al (2012) Cystic fibrosis transmembrane conductance regulator: a molecular model defines the architecture of the anion conduction path and locates a “bottleneck” in the pore. *Biochemistry* 51: 2199–2212 doi:10.1021/bi201888a 22352759 10.1021/bi201888a PMC3316148

[24] Dalton J, Kalid O, Schushan M, Ben-Tal N, Villà-Freixa J (2012) New model of cystic fibrosis transmembrane conductance regulator proposes active channel-like conformation. *J Chem Inf Model* 52: 1842–1853 doi:10.1021/ci2005884 22747419 10.1021/ci2005884

[25] Ward A, Reyes CL, Yu J, Roth CB, Chang G (2007) Flexibility in the ABC transporter MsbA: Alternating access with a twist. *Proc Natl Acad Sci U S A* 104: 19005–19010 doi:10.1073/pnas.0709388104 18024585 10.1073/pnas.0709388104 PMC2141898

[26] Furukawa-Hagiya T, Furuta T, Chiba S, Sohma Y, Sakurai M (2013) The power stroke driven by ATP binding in CFTR as studied by molecular dynamics simulations. *J Phys Chem B* 117: 83–93 doi:10.1021/jp308315w 23214920 10.1021/jp308315w

[27] Aller SG, Yu J, Ward A, Weng Y, Chittaboina S, et al (2009) Structure of P-glycoprotein reveals a molecular basis for poly-specific drug binding. *Science* 323: 1718–1722 doi:10.1126/science.1168750 19325113 10.1126/science.1168750 PMC2720052

[28] Sali A, Blundell TL (1993) Comparative protein modelling by satisfaction of spatial restraints. *J Mol Biol* 234: 779–815 doi:10.1006/jmbi.1993.1626 8254673 10.1006/jmbi.1993.1626

[29] Cotten JF, Welsh MJ (1999) Cystic fibrosis-associated mutations at arginine 347 alter the pore architecture of CFTR. Evidence for disruption of a salt bridge. *J Biol Chem* 274: 5429–5435. 10026154 10.1074/jbc.274.9.5429

[30] Cui G, Zhang Z-R, O’Brien ARW, Song B, McCarty NA (2008) Mutations at arginine 352 alter the pore architecture of CFTR. *J Membr Biol* 222: 91–106 doi:10.1007/s00232-008-9105-9 18421494 10.1007/s00232-008-9105-9 PMC2474774

[31] Zhang Z-R, Zeltwanger S, McCarty NA (2000) Direct comparison of NPPB and DPC as probes of CFTR expressed in *Xenopus* oocytes. *J Membr Biol* 175: 35–52. 10811966 10.1007/s002320001053

[32] McCarty NA, Zhang Z-R (2001) Identification of a region of strong discrimination in the pore of CFTR. *Am J Physiol Lung Cell Mol Physiol* 281: L852–L867. 11557589 10.1152/ajplung.2001.281.4.L852

[33] Smith SS, Liu X, Zhang Z-R, Sun F, Kriewall TE, et al (2001) CFTR: covalent and noncovalent modification suggests a role for fixed charges in anion conduction. *J Gen Physiol* 118: 407–431. 11585852 10.1085/jgp.118.4.407 PMC2233702

[34] Gong X, Linsdell P (2003) Molecular determinants and role of an anion binding site in the external mouth of the CFTR chloride channel pore. *J Physiol* 549: 387–397 doi:10.1113/jphysiol.2002.038232 12679372 10.1113/jphysiol.2002.038232 PMC2342941

[35] Gong X, Burbridge SM, Cowley EA, Linsdell P (2002) Molecular determinants of Au (CN)_2_ ^−^ binding and permeability within the cystic fibrosis transmembrane conductance regulator Cl^−^ channel pore. *J Physiol* 540: 39–47. 11927667 10.1113/jphysiol.2001.013235 PMC2290216

[36] Gupta J, Evagelidis A, Hanrahan JW, Linsdell P (2001) Asymmetric structure of the cystic fibrosis transmembrane conductance regulator chloride channel pore suggested by mutagenesis of the twelfth transmembrane region. *Biochemistry* 40: 6620–6627. 11380256 10.1021/bi002819v

[37] Liu X, Zhang Z-R, Fuller MD, Billingsley J, McCarty NA, et al (2004) CFTR: a cysteine at position 338 in TM6 senses a positive electrostatic potential in the pore. *Biophys J* 87: 3826–3841 doi:10.1529/biophysj.104.050534 15361410 10.1529/biophysj.104.050534 PMC1304894

[38] Aubin CNS, Linsdell P (2006) Positive charges at the intracellular mouth of the pore regulate anion conduction in the CFTR chloride channel. *J Gen Physiol* 128: 535–545 doi:10.1085/jgp.200609516 17043152 10.1085/jgp.200609516 PMC2151590

[39] Beck EJ, Yang Y, Yaemsiri S, Raghuram V (2008) Conformational changes in a pore-lining helix coupled to cystic fibrosis transmembrane conductance regulator channel gating. *J Biol Chem* 283: 4957–4966 doi:10.1074/jbc.M702235200 18056267 10.1074/jbc.M702235200

[40] Fatehi M, Linsdell P (2008) State-dependent access of anions to the cystic fibrosis transmembrane conductance regulator chloride channel pore. *J Biol Chem* 283: 6102–6109 doi:10.1074/jbc.M707736200 18167343 10.1074/jbc.M707736200

[41] Serrano JR, Liu X, Borg ER, Alexander CS, Shaw CF, et al (2006) CFTR: Ligand exchange between a permeant anion ([Au(CN)_2_]^−^) and an engineered cysteine (T338C) blocks the pore. *Biophys J* 91: 1737–1748 doi:10.1529/biophysj.105.078899 16766608 10.1529/biophysj.105.078899 PMC1544293

[42] Bai Y, Li M, Hwang T-C (2010) Dual roles of the sixth transmembrane segment of the CFTR chloride channel in gating and permeation. *J Gen Physiol* 136: 293–309 doi:10.1085/jgp.201010480 20805575 10.1085/jgp.201010480 PMC2931150

[43] Cui G, Song B, Turki HW, McCarty NA (2012) Differential contribution of TM6 and TM12 to the pore of CFTR identified by three sulfonylurea-based blockers. *Pflugers Arch* 463: 405–418 doi:10.1007/s00424-011-1035-1 22160394 10.1007/s00424-011-1035-1

[44] Zhou J-J, Li M-S, Qi J, Linsdell P (2010) Regulation of conductance by the number of fixed positive charges in the intracellular vestibule of the CFTR chloride channel pore. *J Gen Physiol* 135: 229–245 doi:10.1085/jgp.200910327 20142516 10.1085/jgp.200910327 PMC2828907

[45] Qian F, Hiani El Y, Linsdell P (2011) Functional arrangement of the 12^th^ transmembrane region in the CFTR chloride channel pore based on functional investigation of a cysteine-less CFTR variant. *Pflugers Arch* 462: 559–571 doi:10.1007/s00424-011-0998-2 21796338 10.1007/s00424-011-0998-2

[46] Chen EY, Bartlett MC, Loo TW, Clarke DM (2004) The DeltaF508 mutation disrupts packing of the transmembrane segments of the cystic fibrosis transmembrane conductance regulator. *J Biol Chem* 279: 39620–39627 doi:10.1074/jbc.M407887200 15272010 10.1074/jbc.M407887200

[47] Wallin E, Tsukihara T, Yoshikawa S, Heijne GV, Elofsson A (1997) Architecture of helix bundle membrane proteins: An analysis of cytochrome c oxidase from bovine mitochondria. *Protein Sci* 6: 808–815 doi:10.1002/pro.5560060407 9098890 10.1002/pro.5560060407 PMC2144765

[48] Marcus Y (1997) Ion Properties. New York: Marcel Dekker. 259 p.

[49] Linsdell P (2006) Mechanism of chloride permeation in the cystic fibrosis transmembrane conductance regulator chloride channel. *Exp Physiol* 91: 123–129 doi:10.1113/expphysiol.2005.031757 16157656 10.1113/expphysiol.2005.031757

[50] Laskowski RA, MacArthur MW, Moss DS, Thornton JM (1993) PROCHECK: a program to check the stereochemical quality of protein structures. *J Appl Crystallogr* 26: 283–291 doi:10.1107/S0021889892009944

[51] Zhang Z-R, Cui G, Liu X, Song B, Dawson DC, et al (2005) Determination of the functional unit of the cystic fibrosis transmembrane conductance regulator chloride channel. One polypeptide forms one pore. *J Biol Chem* 280: 458–468 doi:10.1074/jbc.M409626200 15504728 10.1074/jbc.M409626200

[52] Zhang Z-R, Song B, McCarty NA (2005) State-dependent chemical reactivity of an engineered cysteine reveals conformational changes in the outer vestibule of the cystic fibrosis transmembrane conductance regulator. *J Biol Chem* 280: 41997–42003 doi:10.1074/jbc.M510242200 16227620 10.1074/jbc.M510242200

[53] Norimatsu Y, Ivetac A, Alexander CS, O'Donnell N, Frye L, et al (2012) Locating a plausible binding site for an open-channel blocker, GlyH-101, in the pore of the cystic fibrosis transmembrane conductance regulator. *Mol Pharmacol* 82: 1042–1055 doi:10.1124/mol.112.080267 22923500 10.1124/mol.112.080267 PMC3502623

[54] Jorgensen WL, Chandrasekhar J, Madura JD, Impey RW, Klein ML (1983) Comparison of simple potential functions for simulating liquid water. *J Chem Phys* 79: 926–935 doi:10.1063/1.445869

[55] Grosman C, Zhou M, Auerbach A (2000) Mapping the conformational wave of acetylcholine receptor channel gating. *Nature* 403: 773–776. 10693806 10.1038/35001586

[56] Hohl M, Briand C, Grütter MG, Seeger MA (2012) Crystal structure of a heterodimeric ABC transporter in its inward-facing conformation. *Nat Struct Mol Biol* 19: 395–402 doi:10.1038/nsmb.2267 22447242 10.1038/nsmb.2267

[57] Cui G, Freeman CS, Knotts T, Prince CZ, Kuang C, et al (2013) Two salt bridges differentially contribute to the maintenance of cystic fibrosis transmembrane conductance regulator (CFTR) channel function. *J Biol Chem* 288: 20758–20767 doi:10.1074/jbc.M113.476226 23709221 10.1074/jbc.M113.476226 PMC3711338

[58] Smart OS, Neduvelil JG, Wang X, Wallace BA, Sansom MSP (1996) HOLE: A program for the analysis of the pore dimensions of ion channel structural models. *J Mol Graph* 14: 354–360 doi:10.1016/S0263-7855(97)00009-X 9195488 10.1016/s0263-7855(97)00009-x

[59] Cystic Fibrosis Mutation Database (n.d.) Cystic Fibrosis Mutation Database. Available: http://www.genet.sickkids.on.ca/cftr/app. Accessed 2012 Dec 7.

[60] Fuller MD, Zhang Z-R, Cui G, McCarty NA (2005) The block of CFTR by scorpion venom is state-dependent. *Biophys J* 89: 3960–3975 doi:10.1529/biophysj.105.060731 16183882 10.1529/biophysj.105.060731 PMC1366962

[61] Kogan I, Ramjeesingh M, Li C, Kidd JF, Wang Y, et al (2003) CFTR directly mediates nucleotide-regulated glutathione flux. *EMBO J* 22: 1981–1989 doi:10.1093/emboj/cdg194 12727866 10.1093/emboj/cdg194 PMC156066

[62] Weng J, Fan KN, Wang W (2012) The conformational transition pathways of ATP-binding cassette transporter BtuCD revealed by targeted molecular dynamics simulation. *PLoS One* 7: e30465 doi:10.1371/journal.pone.0030465 22272354 10.1371/journal.pone.0030465 PMC3260306

[63] St-Pierre J-F, Bunker A, Róg T, Karttunen M, Mousseau N (2012) Molecular dynamics simulations of the bacterial ABC transporter SAV1866 in the closed form. *J Phys Chem B* 116: 2934–2942 doi:10.1021/jp209126c 22339391 10.1021/jp209126c

[64] Csanády L, Nairn AC, Gadsby DC (2006) Thermodynamics of CFTR channel gating: a spreading conformational change initiates an irreversible gating cycle. *J Gen Physiol* 128: 523–533 doi:10.1085/jgp.200609558 17043148 10.1085/jgp.200609558 PMC2151586

[65] Liu X, Dawson DC (2011) Cystic fibrosis transmembrane conductance regulator: temperature-dependent cysteine reactivity suggests different stable conformers of the conduction pathway. *Biochemistry* 50: 10311–10317 doi:10.1021/bi201176q 22014307 10.1021/bi201176q PMC3229303

[66] Khafizov K, Staritzbichler R, Stamm M, Forrest LR (2010) A study of the evolution of inverted-topology repeats from LeuT-fold transporters using AlignMe. *Biochemistry* 49: 10702–10713 doi:10.1021/bi101256x 21073167 10.1021/bi101256x

[67] Kyte J, Doolittle RF (1982) A simple method for displaying the hydropathic character of a protein. *J Mol Biol* 157: 105–132. 7108955 10.1016/0022-2836(82)90515-0

[68] McGuffin LJ, Bryson K, Jones DT (2000) The PSIPRED protein structure prediction server. *Bioinformatics* 16: 404–405. 10869041 10.1093/bioinformatics/16.4.404

[69] Viklund H, Elofsson A (2008) OCTOPUS: improving topology prediction by two-track ANN-based preference scores and an extended topological grammar. *Bioinformatics* 24: 1662–1668. 18474507 10.1093/bioinformatics/btn221

[70] Humphrey W, Dalke A, Schulten K (1996) VMD: visual molecular dynamics. *J Mol Graph* 14: 33–38, 27–28. 10.1016/0263-7855(96)00018-5 8744570

[71] Schriml LM, Dean M (2000) Identification of 18 mouse ABC genes and characterization of the ABC superfamily in *Mus musculus* . *Genomics* 64: 24–31 doi:10.1006/geno.1999.6102 10708515 10.1006/geno.1999.6102

[72] Lomize MA, Lomize AL, Pogozheva ID, Mosberg HI (2006) OPM: orientations of proteins in membranes database. *Bioinformatics* 22: 623–625 doi:10.1093/bioinformatics/btk023 16397007 10.1093/bioinformatics/btk023

[73] Phillips JC, Braun R, Wang W, Gumbart J, Tajkhorshid E, et al (2005) Scalable molecular dynamics with NAMD. *J Comput Chem* 26: 1781–1802 doi:10.1002/jcc.20289 16222654 10.1002/jcc.20289 PMC2486339

[74] Brooks BR, Bruccoleri RE, Olafson BD, States DJ, Swaminathan S, et al (1983) CHARMM: A program for macromolecular energy, minimization, and dynamics calculations. *J Comput Chem* 4: 187–217 doi:10.1002/jcc.540040211

