# Supplementary figures and images for: Modeling the Conformational Changes Underlying Channel Opening in CFTR
Source: PLoS One. 2013 Sep 27;8(9):e74574. doi: 10.1371/journal.pone.0074574 (PMC3785483; doi:10.1371/journal.pone.0074574)

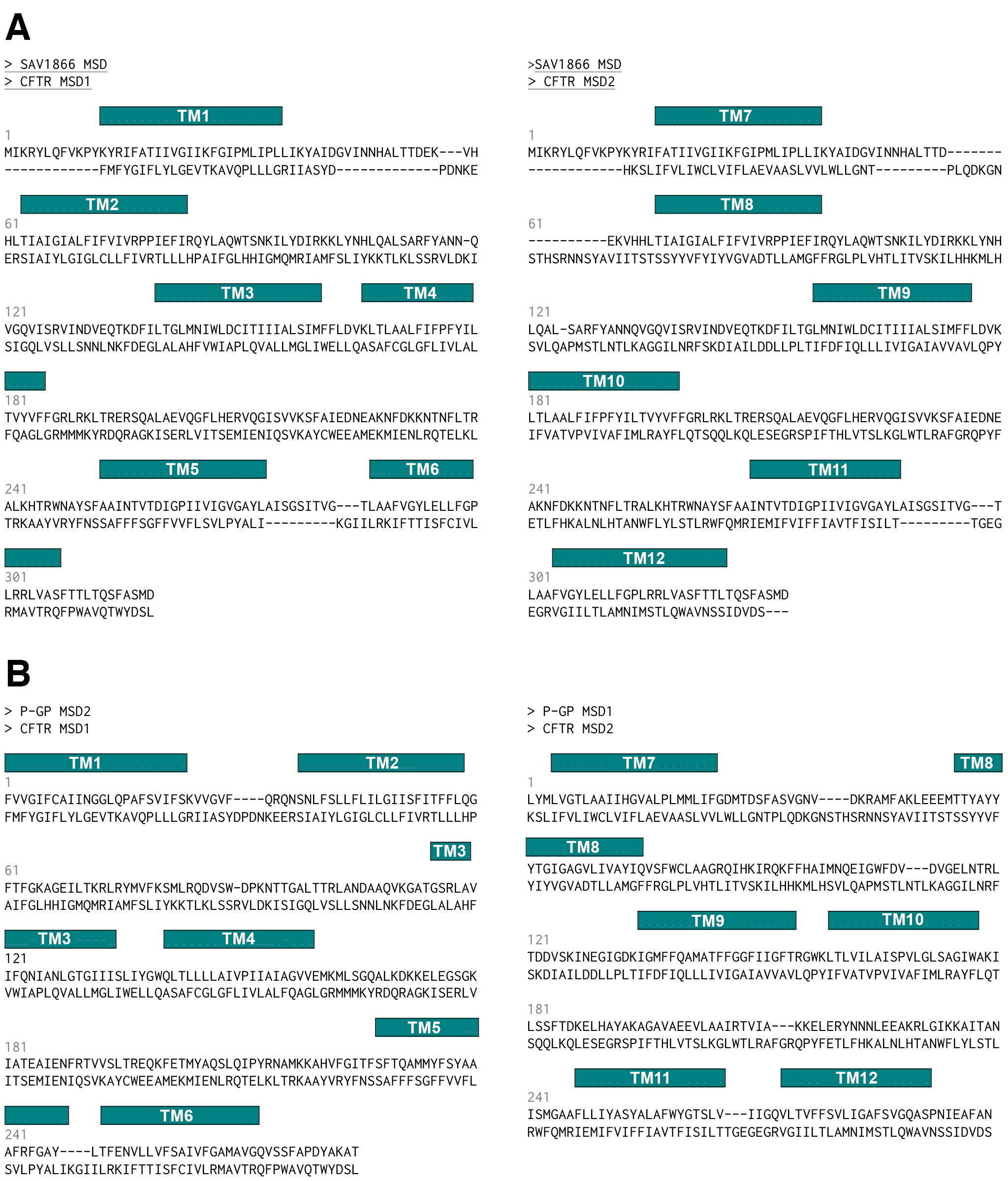

Supplement: Figure S1 — Template Sequence Alignments. Sequence alignments of the membrane-spanning domains of human CFTR with (A) Sav1866 and (B) mouse P-glycoprotein indicating the positions of TM helices. Note that the P-gp MSD2 was aligned with CFTR MSD1 and vice versa. (TIF) [file pone.0074574.s001.tif]

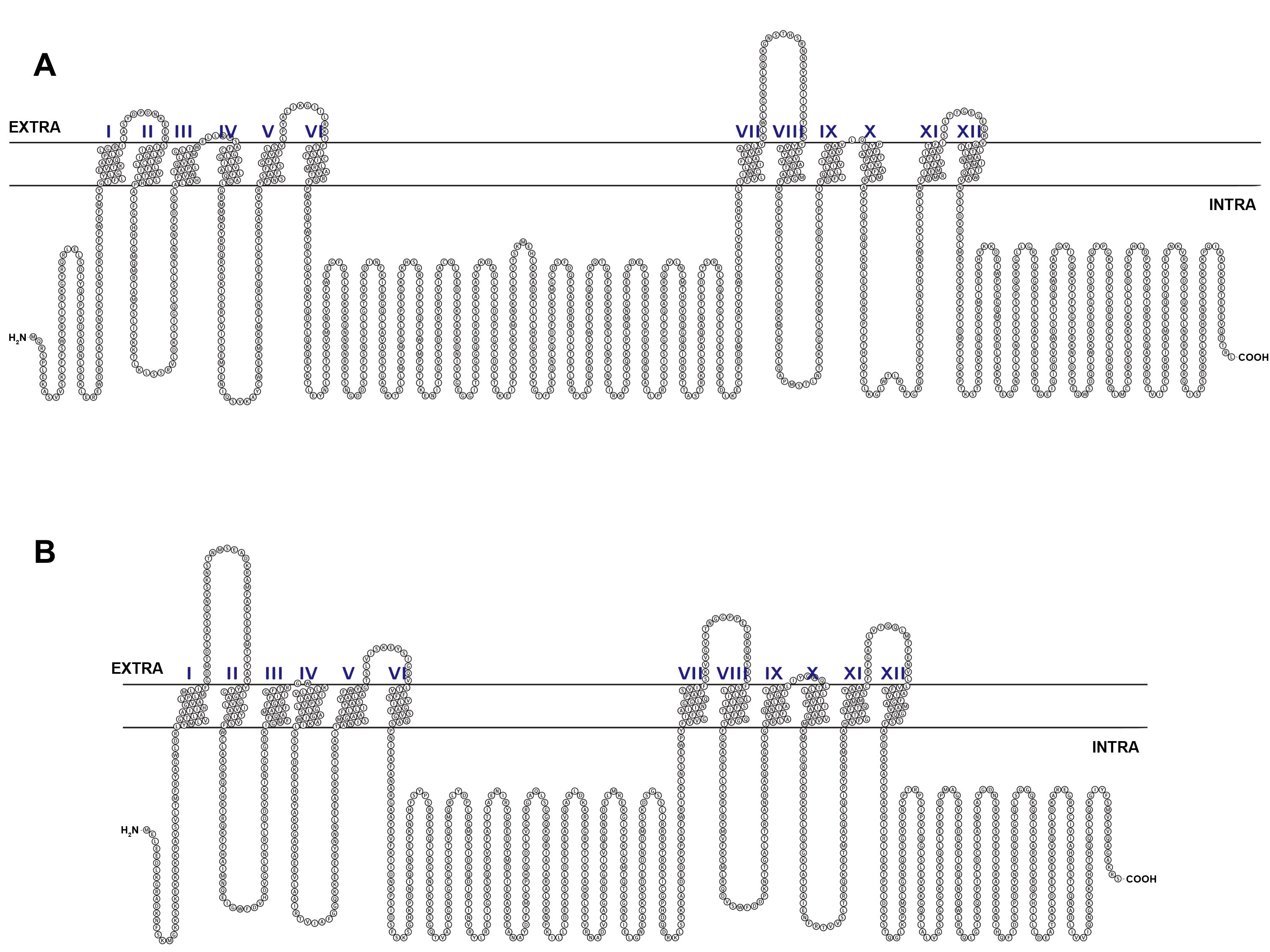

Supplement: Figure S2 — Possible topological swap between CFTR and P-gp. Comparison of the topologies of the O-CFTR model (top) and P-gp (bottom) that demonstrate the discrepancy in the size of the extracellular loops between the two structures. In order to facilitate modeling, MSD1 of P-gp was used as a template for MSD2 of CFTR, and vice versa. (TIF) [file pone.0074574.s002.tif]

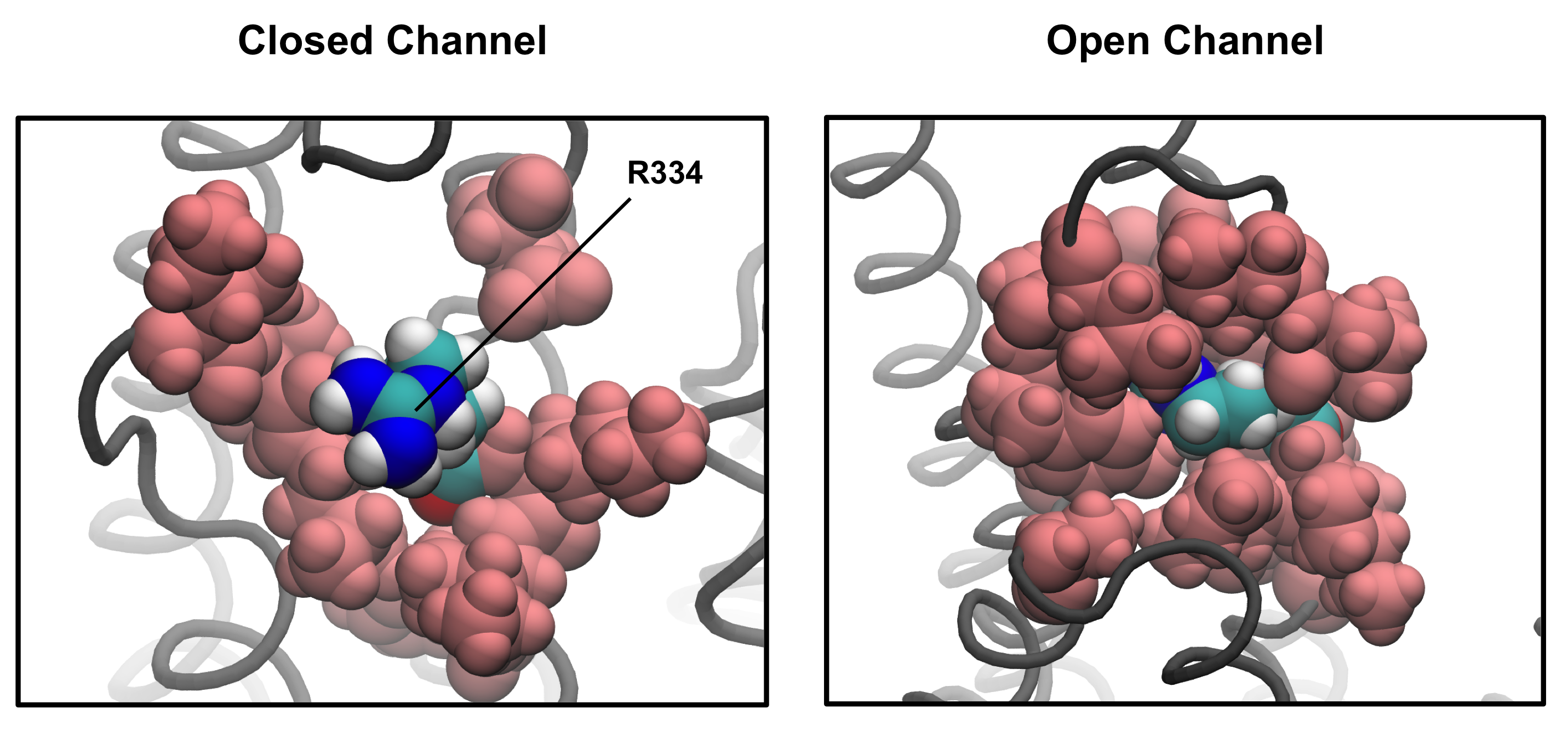

Supplement: Figure S3 — State-dependent accessibility of R334. These images show CFTR from the extracellular side, with the residue R334 represented by spheres in CPK coloring, and all residues with atoms within 5 Å of R334 shown as pink spheres. R334 is largely buried by its neighboring residues in the open channel state, exposing only 7.6% of its surface area to solvent. On the other hand, in the closed channel, R334 is more exposed (57.3% accessible). (TIF) [file pone.0074574.s003.tif]

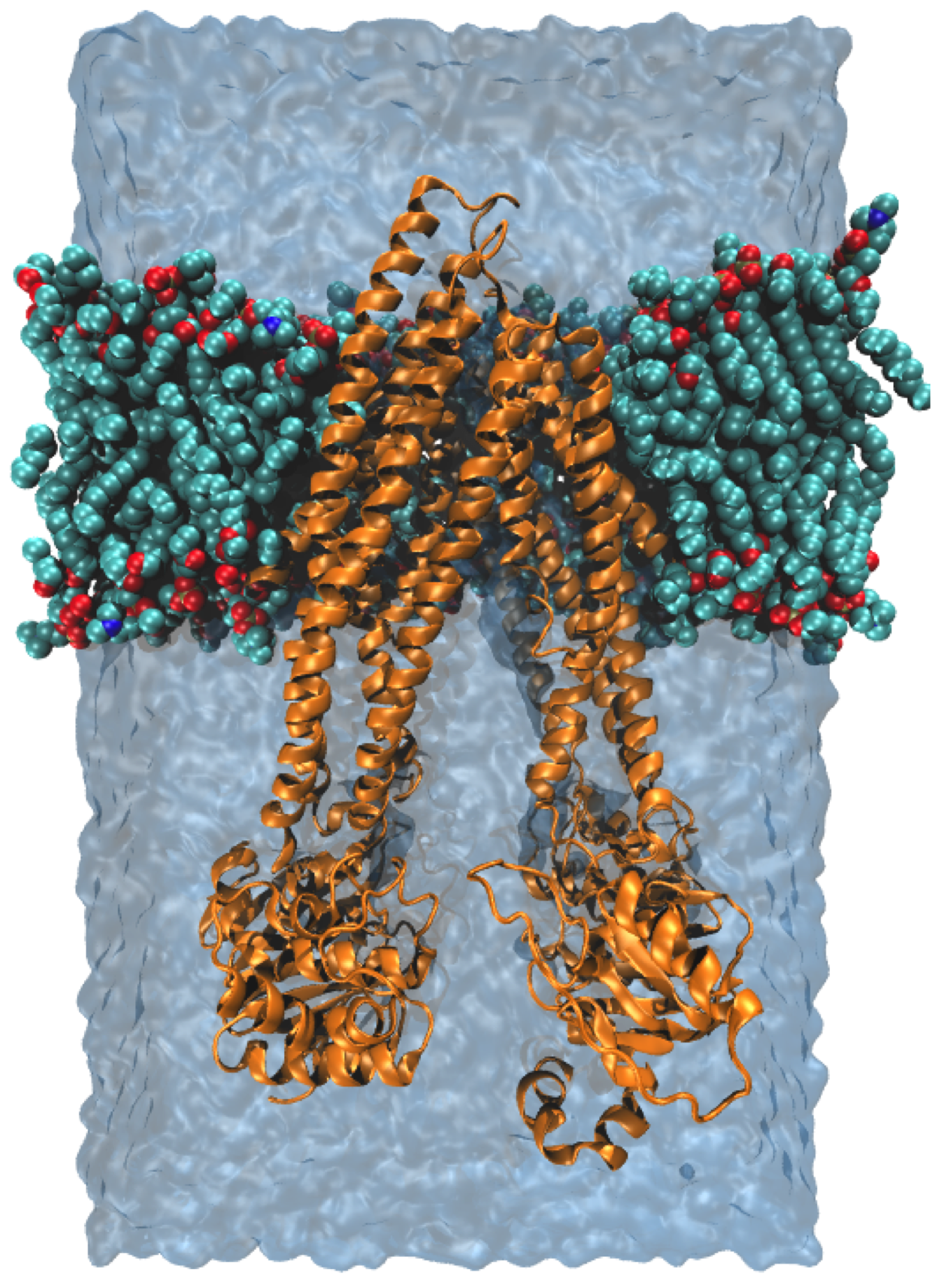

Supplement: Figure S4 — Setting up the simulation. A snapshot of the unit simulation box, showing the C0-CFTR protein model (orange ribbon) embedded in an equilibrated POPC lipid membrane patch (spheres) and enclosed in a water box. Potassium and chloride ions (not shown) are included throughout the solvent. (TIF) [file pone.0074574.s004.tif]

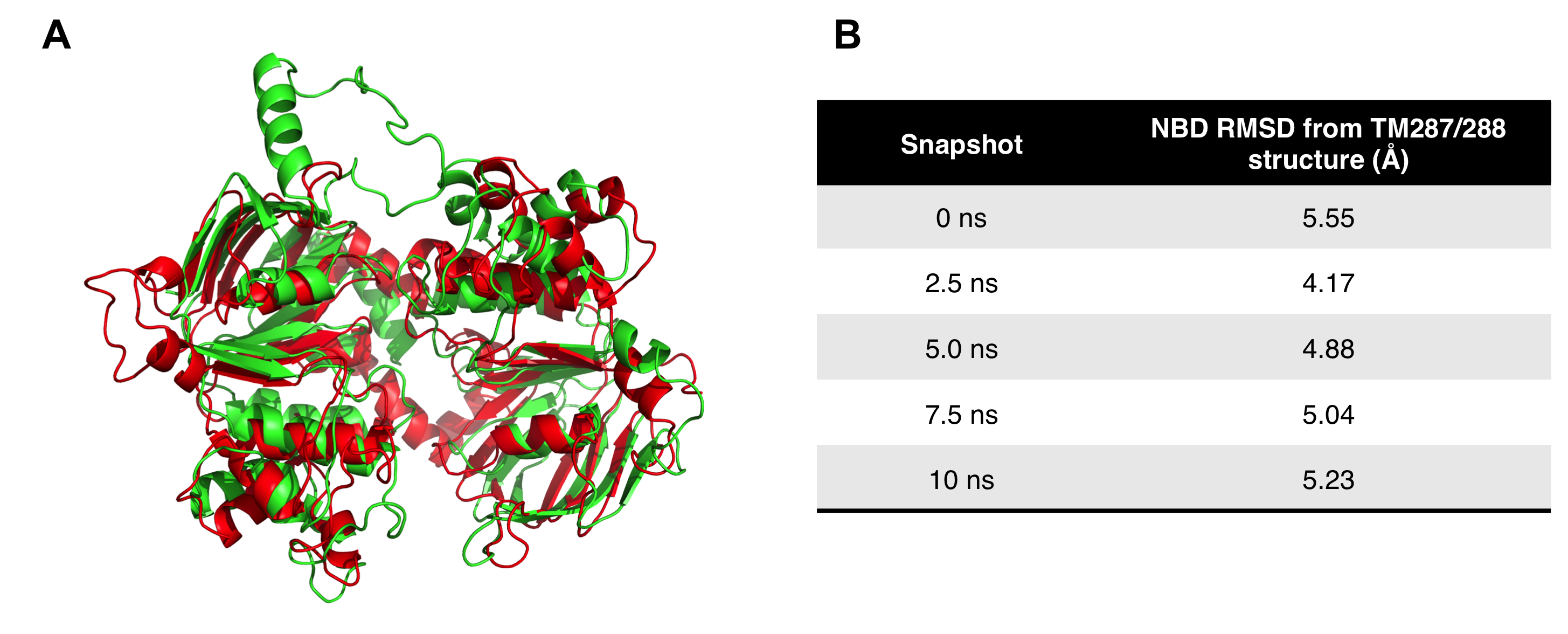

Supplement: Figure S5 — Comparison of NBDs from trajectory structures and TM287/288. PyMOL (Schrödinger, LLC) was used to align, superimpose, and calculate the RMSD between NBDs from the 0 ns, 2.5 ns, 5 ns, 7.5 ns and 10 ns snapshots of the CFTR TMD trajectory with the NBDs from the asymmetric crystal structure of TM287/288 [5]. (A) Image showing superimposed NBDs from the 2.5 ns snapshot (green) and TM287/288 (red). (B) Calculated RMSDs between the NBDs from various trajectory structures and TM287/288. The 2.5 ns snapshot structure most closely resembles the TM287/288 partial dimer structure. (TIF) [file pone.0074574.s005.tif]

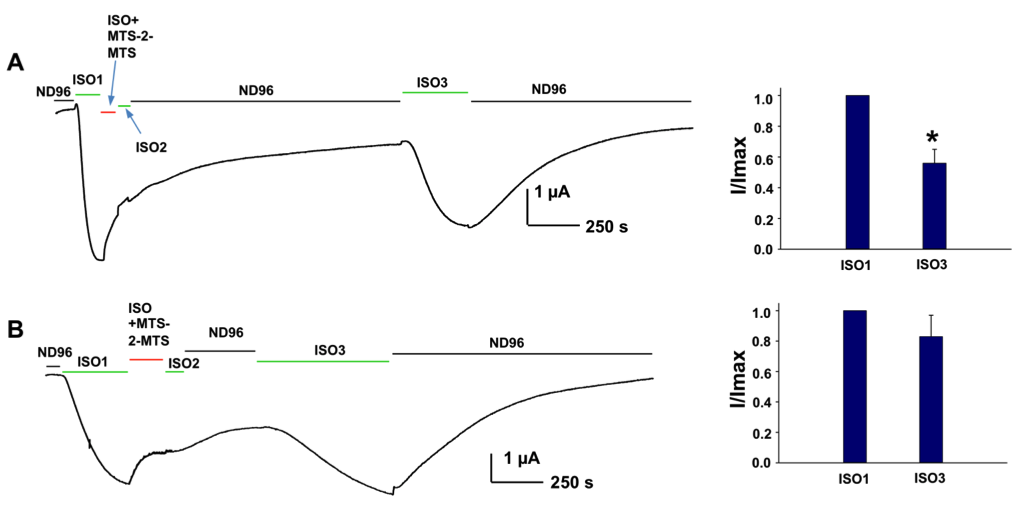

Supplement: Figure S6 — Effects of 1 mM MTS2-2MTS on R334C-CFTR and E217C-CFTR channels. Representative traces (left) and summary data (right) for macroscopic currents measured from R334C- (A) and E217C-CFTR (B) by two-electrode voltage clamp. Channels were activated by exposure of the oocyte to isoproterenol (ISO). ND96 = control bath solution. At this concentration, MTS-2-MTS acts as a pore-blocker, reducing the current levels of both mutants. MTS-2-MTS also appeared to covalently decrease macroscopic current at R334C-CFTR, but not E217C-CFTR, perhaps due to alteration of charge or side-chain volume. However, the bifunctional MTS reagent was not capable of covalently locking closed CFTR channels bearing a single cysteine, unlike its effect on channels bearing cysteines at both positions (Figure 7). Current levels in the summary data are given relative to control conditions prior to first exposure to ISO, and are normalized to maximal current in the response to ISO1 (Imax). *, p<0.05 compared to ISO1. n = 3 for both mutants. (TIF) [file pone.0074574.s006.tif]

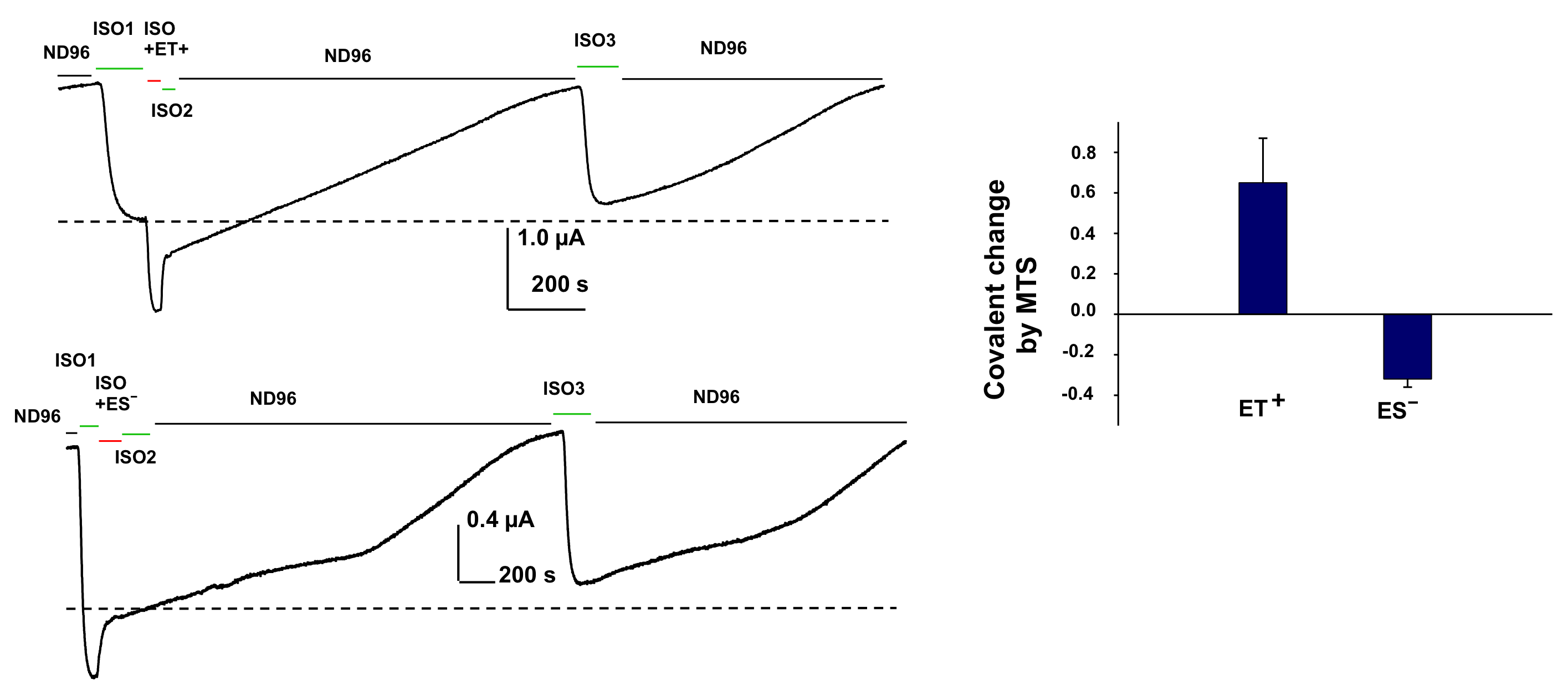

Supplement: Figure S7 — Effects of MTSET (ET+) and MTSES (ES-) on R334C/E217C-CFTR channels. Representative traces (left) and summary data (right) for macroscopic currents measured from R334C/E217C-CFTR by two-electrode voltage clamp with addition of the 1 mM monofunctional MTS reagents MTSET+ (ET+) or MTSES− (ES−) in the presence of isoproterenol (ISO). ND96 = control bath solution. Both ET+ and ES− covalently bound to R334C/E217C-CFTR. MTSET+ led to an increase in current, part of which decayed upon washout of reagent and thus was not covalent. MTSES− led to a decrease in current, all of which appeared to be covalent. Importantly, neither MTSET+ nor MTSES− inhibited the subsequent activation by ISO. Current changes in response to ISO are given relative to control conditions prior to first exposure to ISO1 (IISO1), and current changes by covalent modification by MTS reagents are given relative to the maximal current in response to ISO1(IMTS). Covalent change by MTS = IMTS/IISO1. n = 4 experiments each. (TIF) [file pone.0074574.s007.tif]

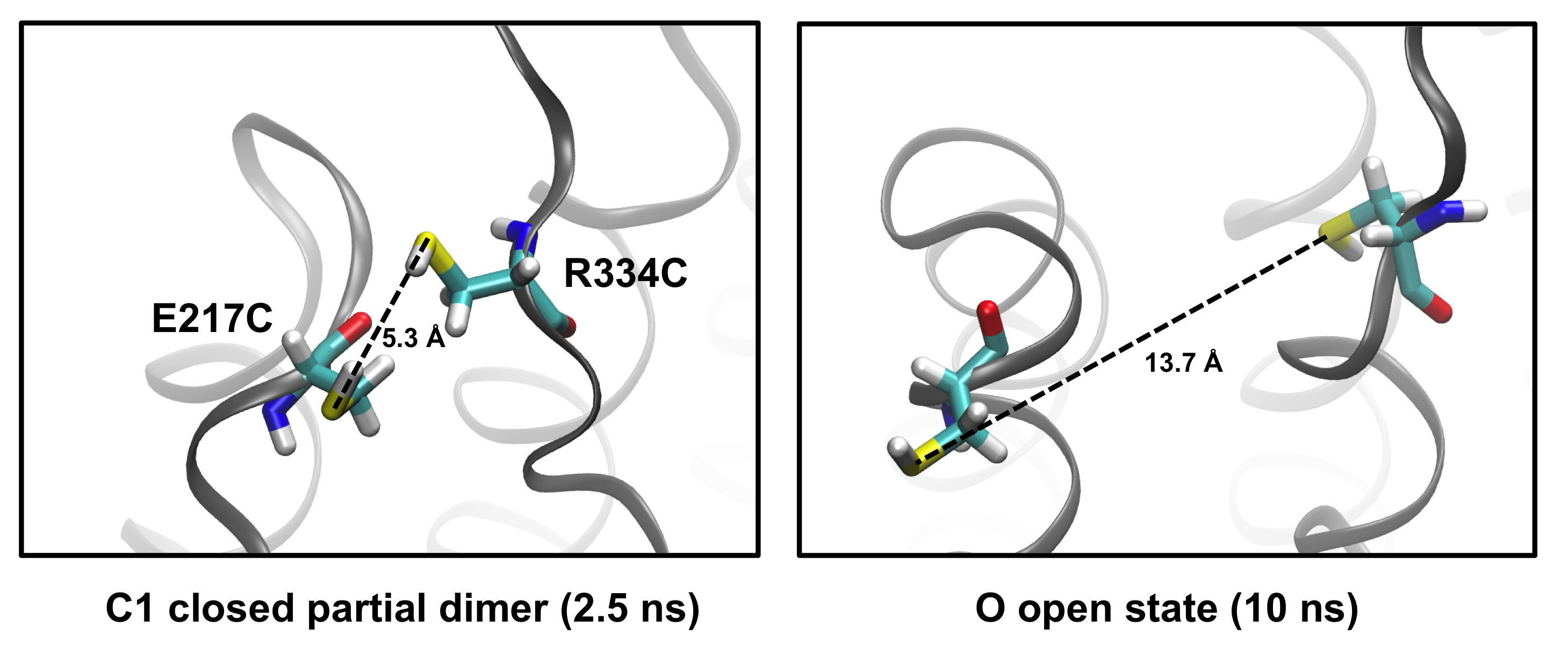

Supplement: Figure S8 — Distances between residues in R334C-E217C Double Mutant. PyMOL (Schrödinger, LLC) was used to mutate residues R334 and E217 to cysteines in the 2.5 ns (C1 partial dimer closed) and 10 ns (O open) snapshots. The distance between the side-chain sulfhydryl groups in the C1 state (2.5 ns) was found to be 5.3 Å, and in the O state (10 ns) snapshot it was 13.7 Å. (TIF) [file pone.0074574.s008.tif]

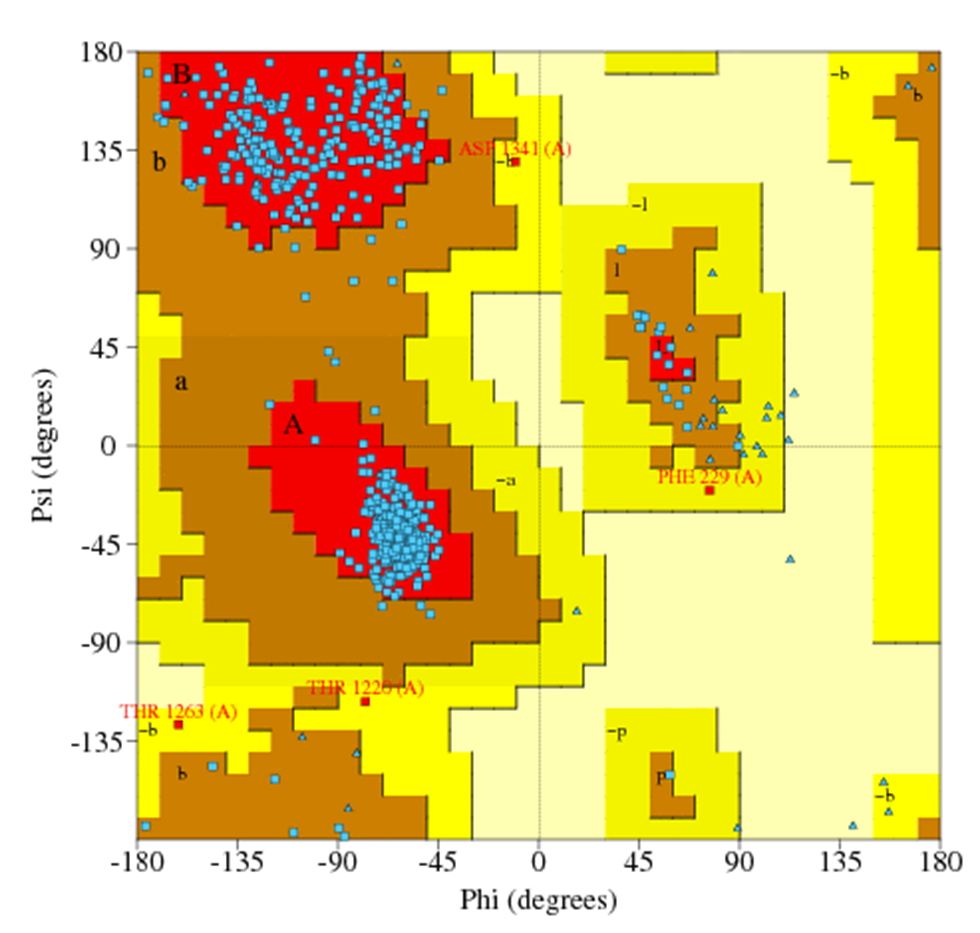

Supplement: Figure S9 — Ramachandran plot of the O-CFTR model. Analysis using PROCHECK [6] reveals 94.6% (910 of 962) non-glycine and non-proline residues in the most favorable "core" regions of the Ramachandran plot (colored red). Of the remainder, 4.6% (44 residues) were found in "additional allowed" regions (orange), and four residues (labeled) were in "generously allowed regions". No residues in O-CFTR were found in disallowed regions of the Ramachandran plot. Glycine residues are plotted as triangles, and all others as squares. (TIF) [file pone.0074574.s009.tif]

## Table S2: Side-chain interactions


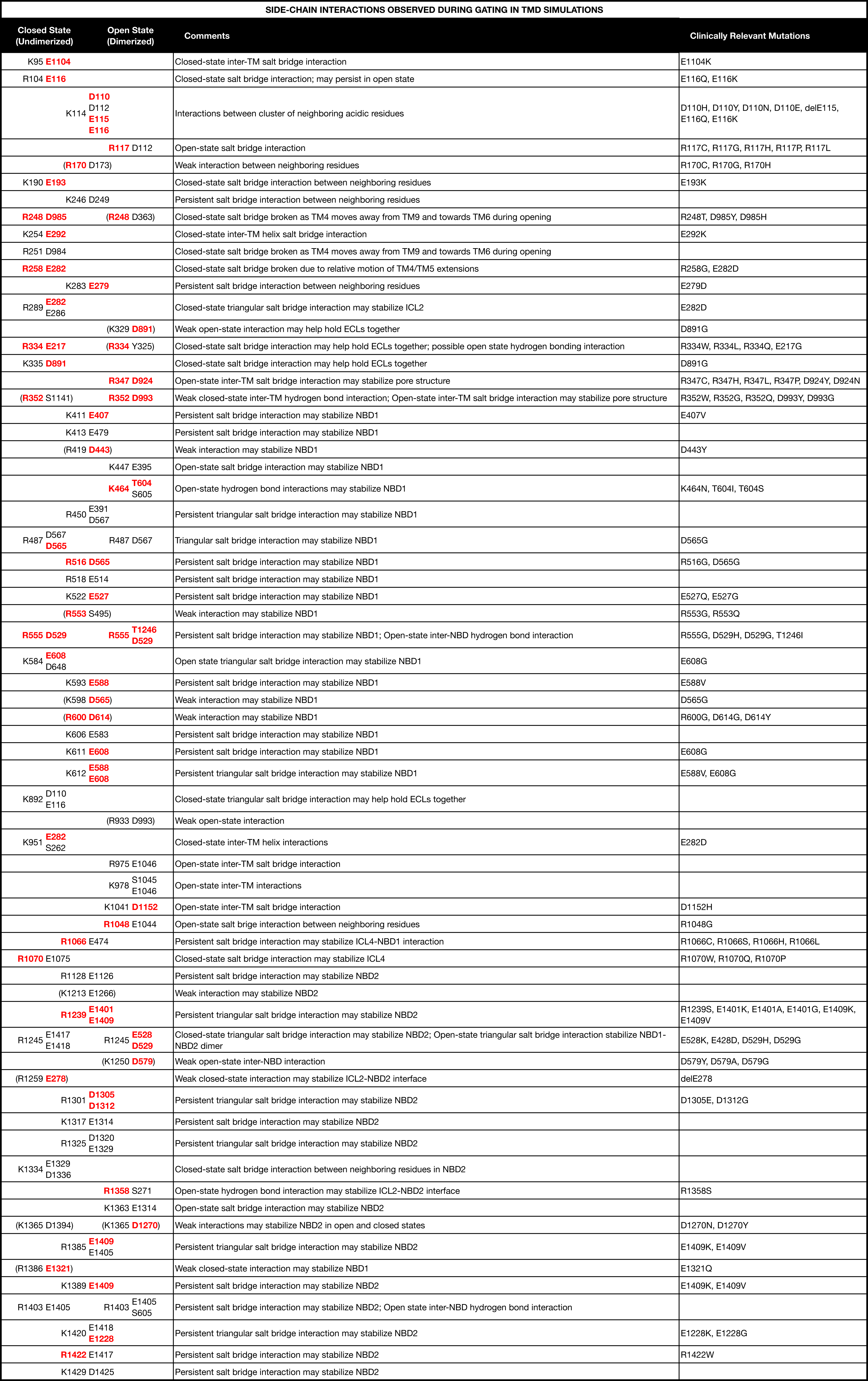

Supplement: Table S2 — Side-chain interactions. (DOCX) [file pone.0074574.s011.docx]
