# Supplementary material for: Modeling the Conformational Changes Underlying Channel Opening in CFTR
Source: PLoS One. 2013 Sep 27;8(9):e74574. doi: 10.1371/journal.pone.0074574 (PMC3785483; doi:10.1371/journal.pone.0074574)
Supplement: Table S1 — Homology modeling distance restraints. (DOCX) [file pone.0074574.s010.docx]

## Table S1: Homology Modeling Distance Restraints

| **Residues** | **Distance** | **Reference** |
| --- | --- | --- |
| R352-D993 | ≤ 5 Å (salt bridge) | [1] |
| R347-D924 | ≤ 5 Å (salt bridge) | [2] |
| M348-T1142 | ≤ 13 Å | [3] |
| T351-T1142 | 9 Å ≤ *d* ≤ 13 Å | [3] |
| S605-A1374 | ≤ 8 Å | [4] |
| S549-S1248 | ≤ 8 Å | [4] |
| S459-V1379 | ≤ 8 Å | [4] |
| S434-D1336 | 8 Å ≤ *d* ≤ 16 Å | [4] |
| S549-A1374 | 8 Å ≤ *d* ≤ 16 Å | [4] |
