# Supplementary material for: Modeling the Conformational Changes Underlying Channel Opening in CFTR
Source: PLoS One. 2013 Sep 27;8(9):e74574. doi: 10.1371/journal.pone.0074574 (PMC3785483; doi:10.1371/journal.pone.0074574)
Supplement: References S1 — List of references cited in other Supporting Information. (DOCX) [file pone.0074574.s012.docx]

# SUPPORTING INFORMATION References

1. Cui G, Zhang Z-R, O’Brien ARW, Song B, McCarty NA (2008) Mutations at arginine 352 alter the pore architecture of CFTR. *J Membr Biol* **222**: 91–106. doi:10.1007/s00232-008-9105-9.

2. Cotten JF, Welsh MJ (1999) Cystic fibrosis-associated mutations at arginine 347 alter the pore architecture of CFTR. Evidence for disruption of a salt bridge. *J Biol Chem* **274**: 5429–5435.

3. Chen EY, Bartlett MC, Loo TW, Clarke DM (2004) The DeltaF508 mutation disrupts packing of the transmembrane segments of the cystic fibrosis transmembrane conductance regulator. *J Biol Chem* **279**: 39620–39627. doi:10.1074/jbc.M407887200.

4. Mense M, Vergani P, White DM, Altberg G, Nairn AC, et al. (2006) In vivo phosphorylation of CFTR promotes formation of a nucleotide-binding domain heterodimer. *EMBO J* **25**: 4728–4739. doi:10.1038/sj.emboj.7601373.

5. Hohl M, Briand C, Grütter MG, Seeger MA (2012) Crystal structure of a heterodimeric ABC transporter in its inward-facing conformation. *Nat Struct Mol Biol* **19**: 395–402. doi:10.1038/nsmb.2267.

6. Laskowski RA, MacArthur MW, Moss DS, Thornton JM (1993) PROCHECK: a program to check the stereochemical quality of protein structures. *J Appl Crystallogr* **26**: 283–291. doi:10.1107/S0021889892009944.
